# Testing the homogeneity of odds ratio across strata for combined bilateral and unilateral data

**DOI:** 10.1371/journal.pone.0307276

**Published:** 2024-07-18

**Authors:** Shuangcheng Hua, Changxing Ma

**Affiliations:** Department of Biostatistics, University at Buffalo, Buffalo, New York, United States of America; Local Health Authority Caserta: Azienda Sanitaria Locale Caserta, ITALY

## Abstract

Bilateral and unilateral combined data are commonly involved in clinical trials or observational studies designed to test the treatment effectiveness on paired organs or bodily parts within individual subjects. It is essential to examine if the treatment effect is consistent across different subgroups such as age, gender, or disease severity for understanding how the treatment works for various patient populations. In this paper, we propose three large-sample homogeneity tests of odds ratio in the stratified randomization setting using correlated combined data. Our simulation results show that the score test exhibits robust empirical type I error control and demonstrates strong power characteristics compared to other methods proposed. We apply the proposed tests to real-world datasets of acute otitis media and myopia to illustrate their practical application and utility.

## Introduction

Bilateral data are commonly encountered in clinical trials or observational studies designed to investigate the effectiveness of a novel medicine or treatment on paired organs or bodily parts. For instance, in the context of an ophthalmologic study, it is well recognized that the bilateral measurements collected from both the right and left eyes of a person are correlated and can mutually influence one another. In the case of bilateral data, ignoring the variation inflation caused by correlation within the subject will cause inflated type I error. Hence, it is imperative to take into account the presence of data dependency in standard statistical tests, as the assumption of independence among observations does not hold true. Moreover, it is acknowledged that challenges in collecting complete data from both eyes of a patient may arise due to factors such as patient anxiety, disabilities, or other limiting conditions in clinical practice. It is noteworthy that in situations where data collection can occur bilaterally or unilaterally, neglecting to account for either scenario will lead to diminished statistical power while conducting statistical inference.

In light of the nature of bilateral data in this study, multiple models can be employed to assess the intraclass correlation between the observations obtained from an individual. Donner [1] proposed a method based on an adjusted Pearson chi-square test for the homogeneity of proportions that intuitively assumes a shared intraclass correlation *ρ* among groups, and its validity has been subsequently evaluated [2]. Other methods include Rosner [3] who proposed a constant R model assuming that the conditional probability of a pair of bilateral responses is proportional to the event rate, and Dallal [4] who criticized Rosner’s model due to its poor fit when applied to groups with varying event rates. Westgate [5] estimated intraclass correlation by fitting a GEE-type marginal model in the setting of clustered randomized trials. Cluster bootstrapping [6] is a non-parametric method that estimates standard error within correlated data, however, it is computationally intensive. In this article, we adopt Donner’s *ρ* model due to its robustness in handling bilateral data. In addition, we focus on stratified randomization design as its advantages in preventing imbalances on baseline characteristics between groups. For example, in ophthalmologic studies, factors such as age, severity of the eye condition are known to influence the treatment effectiveness, thus introducing bias into the outcomes. In the context of stratified randomization in clinical trials, it is of common interest to investigate the treatment effects by comparing the proportions observed in each group, and assess the uniformity of treatment effects across different strata.

Specifically, this study aims to develop statistical procedures for testing the homogeneity of treatment effect, as measured by odds ratio, using both bilateral and unilateral data across strata. The decision to prioritize odds ratio as the measure of treatment effect is motivated by its frequent usage in observational studies, such as case-control studies, where the prevalence of the outcome can not be estimated. In addition, the odds ratio is commonly utilized in randomized controlled trials, particularly when a logistic regression model is employed. Previous research has investigated various exact and asymptotic homogeneity tests of odds ratios, such as Breslow–Day, DerSimonian-Laird, Pearson chi-square, Zelen, score test, among others, in *K* 2 × 2 tables [7–12]. Rather than test-based approaches, logistic regression model-based methods are utilized to assess the homogeneity of odds ratio [13–15]. Kulinskaya [16] introduced a test based on Cochran’s Q-statistics in meta-analysis of *K* independent studies. For correlated data, Song [17] proposed modified Breslow–Day, Tarone and conditional score test under clustered randomized trials. Nevertheless, these studies focus on either independent data or correlated data. Our methodologies are designed for homogeneity tests of odds ratio accommodating both independent unilateral and correlated bilateral data.

The scope of contributions made by this work extends beyond the following: (i) While multiple existing studies have been conducted on homogeneity tests of odds ratio, there is still a dearth of research addressing correlated bilateral data under stratified randomization setting. In fact, as pointed out by [18], a majority of ophthalmologic studies analyze eye data at the ocular level rather than treating the individual as a whole with correlated bilateral organs (e.g., [19, 20]). Ignoring such intereye correlation will result in inflated type I error rates. (ii) Our methodologies encompass a wider range of situations to handle both independent unilateral and correlated bilateral data in clinical practice. With that being said, either the bilateral data alone or unilateral data alone constitutes a special case within the framework of our study. The rest of this article is organized as follows. In methods section, we structure the stratified bilateral and unilateral data setting and propose the three large-sample tests along with the derivation of their maximum likelihood estimation procedures. The Monte Carlo simulation section evaluates the performance of the proposed testing methods. In real data examples section, we apply the proposed methods to a study on acute otitis media and another recent study on myopia. We end with a discussion.

## Methods

The purpose of this section is to establish statistical testing methods for assessing the homogeneity of the odds ratio *θ* across different strata. This section is comprised of three parts. The initial part involves the introduction of the bilateral and unilateral combined data structures. Subsequently, we proceed to derive the unconstrained maximum likelihood estimators (MLEs) for the targeting parameters and the constrained MLEs under the null hypothesis of homogeneity. In the third part, we present a framework for three large-sample testing methods based on the maximum likelihood estimators obtained.

### Data design and model

Consider a clinical trial in which *M* individuals have bilateral data and *N* participants have unilateral data. To ensure equal allocation of subgroups of participants to each experimental condition, we divide participants into *J* strata to eliminate potential confounder effect. Within each stratum, participants are randomly assigned to the treatment and control groups. As shown in Table 1, for unilateral data, let *n*_*tij*_ denote the number of participants in the *j*^*th*^ (*j* ∈ {1, …, *J*}) stratum from the *i*^*th*^ (*i* = 1 treatment; *i* = 2 control) group with *t* (*t* ∈ {0, 1}) eyes in the expected event (e.g., cure, disease improvement, etc.), and similarly for bilateral data, let *m*_*tij*_ represent the number of participants in the *j*^*th*^ stratum from the *i*^*th*^ group with *t* (*t* ∈ {0, 1, 2}) eyes in the expected response.

**Table 1:** Frequencies of the individuals within the *j*^*th*^ stratum under stratified bilateral and unilateral combined data.

	Unilateral			Bilateral		
Number of positive responses	Treatment	Control	Total	Treatment	Control	Total
0	*n*_01*j*_	*n*_02*j*_	*n*_0+*j*_	*m*_01*j*_	*m*_02*j*_	*m*_0+*j*_
1	*n*_11*j*_	*n*_12*j*_	*n*_1+*j*_	*m*_11*j*_	*m*_12*j*_	*m*_1+*j*_
2	-	-	-	*m*_21*j*_	*m*_22*j*_	*m*_2+*j*_
Total	*n*_+1*j*_	*n*_+2*j*_	*N*_*j*_	*m*_+1*j*_	*m*_+2*j*_	*M*_*j*_

We define *Z*_*ijkt*_ as a binary response variable with *Z*_*ijkt*_ = 1 indicating that the *t*^*th*^ (*t* = 1 under the unilateral setting and *t* ∈ {1, 2} under the bilateral setting) eye of the *k*^*th*^ (*k* ∈ {1, …, *K*}) patient in the *j*^*th*^ (*j* ∈ {1, …, *J*}) stratum from the *i*^*th*^ (*i* ∈ {1, 2}) group gives an expected event at the end of the trial. To capture the intraclass correlation between two eyes of each individual, we adopt the parametric model proposed by Donner [1] given as follows:

$$Pr\left(Z_{ijkt}=1\right)=\pi_{ij};$$

$$Corr\left(Z_{ijk1},Z_{ijk2}\right)=\rho_{j},\;0\leq \rho_{j}\leq 1,$$

where *π*_*ij*_ represents the probability of the expected event in the *j*^*th*^ stratum from the *i*^*th*^ group, and *ρ*_*j*_ represents the correlation between the paired eyes of each individual if bilateral data are provided. It is worth noting that we generalize Donner’s model to accommodate the stratification by assuming equal correlation between groups but different across strata, which eliminates potential bias between groups and provides variability among strata at the same time.

For unilateral data, it is intuitive that *n*_1*ij*_ ∼ *Binomial*(*n*_+*ij*_, *π*_*ij*_), while as for bilateral data, (*m*_0*ij*_, *m*_1*ij*_, *m*_2*ij*_) ∼ *Multinominal*(*m*_+*ij*_; *p*_0*ij*_, *p*_1*ij*_, *p*_2*ij*_). After incorporating Donner’s model, we have *p*_0*ij*_ = (1 − *π*_*ij*_)(*ρ*_*j*_*π*_*ij*_ − *π*_*ij*_ + 1), *p*_1*ij*_ = 2*π*_*ij*_(1 − *π*_*ij*_)(1 − *ρ*_*j*_) and *p*_2*ij*_ = *π*_*ij*_(*ρ*_*j*_ + *π*_*ij*_ − *ρ*_*j*_*π*_*ij*_) indicating that the probability that a subject in the *j*^*th*^ stratum from the *i*^*th*^ group makes improvement for none, one, and both eyes at the end of the trial.

### Maximum likelihood estimator

Assuming independence among subjects with either bilateral or unilateral data, the log-likelihood function *l*(*π*_1_, *π*_2_, *ρ*) under stratification can be expressed as:

$$l\left(\pi_{1},\pi_{2},\rho \right)=\sum_{j=1}^{J}l_{j}\left(\pi_{1j},\pi_{2j},\rho_{j}\right)+\text{log}\;C,$$

where

$$\begin{array}{ll} l_{j}\left(\pi_{1j},\pi_{2j},\rho_{j}\right) & =\overset{2}{\underset{i=1}{\sum }}\left[n_{0ij}\;\text{log}\left(1-\pi_{ij}\right)\right)+n_{1ij}\;\text{log}\pi_{ij}+m_{0ij}\;\text{log}\left(\left(1-\pi_{ij}\right)\left(\rho \pi_{ij}-\pi_{ij}+1\right)\right)\\ & +m_{1ij}\;\text{log}\left(2\pi_{ij}\left(1-\pi_{ij}\right)\left(1-\rho_{j}\right)\right)+m_{2ij}\;\text{log}\left(\pi_{ij}\left(\rho_{j}+\pi_{ij}-\rho_{j}\pi_{ij}\right)\right)]. \end{array}$$

#### Unconstrained MLEs

First of all, we derive the maximum likelihood estimators of *π*_*ij*_, *ρ*_*j*_ and *θ*_*j*_ under no constraints. Differentiating the log-likelihood function *l*_*j*_(*π*_1*j*_, *π*_2*j*_, *ρ*_*j*_) with respect to *π*_*ij*_, *ρ*_*j*_ yields:

$$\begin{array}{cc} \frac{\partial l_{j}}{\partial \pi_{ij}} & =\frac{n_{0ij}}{\pi_{ij}-1}+\frac{n_{1ij}}{\pi_{ij}}-\frac{m_{0ij}\;(2\;\pi_{ij}-2\rho_{j}\;\pi_{ij}\;+\rho_{j}-2)}{\left(\pi_{ij}-1)\;(\rho_{j}\pi_{ij}-\pi_{ij}+1\right)}\\ & +\frac{m_{1ij}\;(2\;\pi_{ij}-1)}{\pi_{ij}\;(\pi_{ij}-1)}+\frac{m_{2ij}\;(2\;\pi_{ij}-2\rho \;\pi_{ij}\;+\rho_{j})}{\pi_{ij}\;(\pi_{ij}-\rho_{j}\pi_{ij}\;+\rho_{j})}, \end{array}$$ (1)

$$\frac{\partial l_{j}}{\partial \rho_{j}}=\sum_{i=1}^{2}(\frac{m_{0ij}\pi_{ij}}{\rho_{j}\pi_{ij}-\pi_{ij}+1}+\frac{m_{1ij}}{\rho_{j}-1}-\frac{m_{2ij}\left(\pi_{ij}-1\right)}{\pi_{ij}-\rho_{j}\pi_{ij}+\rho_{j}}).$$ (2)

It is worth noting that setting the derivative Eq (1) to be 0 results in a third-degree polynomial of *π*_*ij*_ with close-form solution given a fixed value of *ρ*_*j*_ simplified by [21] as follows:

$$\begin{array}{c} \pi_{ij}=\frac{b_{ij}-\sqrt{b_{ij}^{2}-3c_{ij}\left(2M_{j}+N_{j}\right)}\left(\text{cos}\left(d_{ij}\right)+\sqrt{3}\;\text{sin}\;\left(d_{ij}\right)\right)}{\left(2M_{j}+N_{j}\right)\left(3\rho_{j}-3\right)}, \end{array}$$ (3)

where

$$\begin{array}{l} b_{ij}=\rho_{j}(3M_{j}+2N_{j}+n_{1+j})-2m_{0ij}-3m_{1ij}-4m_{2ij}-n_{0ij}-2n_{1ij},\\ c_{ij}=\rho_{j}^{2}(M_{j}+n_{1+j})-\rho_{j}(2m_{0ij}+4m_{1ij}+4m_{2ij}+n_{0ij}+3n_{1ij})+m_{1+j}+2m_{2+j}+n_{1+j},\\ d_{ij}=\frac{1}{3}\text{arccos}\left(\frac{2b_{ij}^{3}-9(2M_{j}+N_{j})b_{ij}c_{ij}+27{\left(2M_{j}+N_{j}\right)}^{2}(m_{1+j}+m_{2+j}+n_{1+j})(1-\rho_{j})\rho_{j}}{2\sqrt{{\left(b_{ij}^{2}-3c_{ij}(2M_{j}+N_{j})\right)}^{3}}}\right). \end{array}$$

With aforementioned, we incorporate the polynomial function in the Fisher Scoring method to reduce the dimension of Fisher information matrix. The unconstrained MLEs of *π*_*ij*_, *ρ*_*j*_ and *θ*_*j*_ are generated, denoted by ${\hat{\pi }}_{ij}$, ${\hat{\rho }}_{j}$ and ${\hat{\theta }}_{j}$, respectively. Our proposed algorithm 1 described below is faster and exhibits superior robustness than the traditional Fisher scoring method.

**Algorithm 1 Fisher Scoring method for** ${\hat{\pi }}_{ij}$ , ${\hat{\rho }}_{j}$ **and** ${\hat{\theta }}_{j}$

1. Set initial value of $\rho_{j}^{\left(0\right)}=\text{Unif}\left(0,1\right)$.

2. For *t* = (0, 1, …) until convergence:

 (a) Calculate $\pi_{ij}^{\left(t\right)}$ based on Eq (3).

 (b) Update $\rho_{j}^{\left(t+1\right)}$ based on

$$\rho_{j}^{\left(t+1\right)}=\rho_{j}^{\left(t\right)}+{\left[I\left(\rho_{j}|\pi_{ij}^{\left(t\right)},\rho_{j}^{\left(t\right)}\right)\right]}^{-1}\frac{\partial l_{j}}{\partial \rho_{j}}\left(\pi_{ij}^{\left(t\right)},\rho_{j}^{\left(t\right)}\right),$$

 where

$$I\left(\rho_{j}\right)=\sum_{i=1}^{2}(\frac{m_{+ij}\pi_{ij}^{2}\left(1-\pi_{ij}\right)}{\rho_{j}\pi_{ij}-\pi_{ij}+1}+\frac{2m_{+ij}\pi_{ij}\left(1-\pi_{ij}\right)}{1-\rho_{j}}+\frac{m_{+ij}\pi_{ij}^{2}{\left(1-\pi_{ij}\right)}^{2}}{\rho_{j}\pi_{ij}\left(1-\pi_{ij}\right)+\pi_{ij}^{2}}).$$

3. Calculate ${\hat{\theta }}_{j}=\frac{{\hat{\pi }}_{1j}\left(1-{\hat{\pi }}_{2j}\right)}{{\hat{\pi }}_{2j}\left(1-{\hat{\pi }}_{1j}\right)}$.

#### Constrained MLEs under homogeneity

Followed by unconstrained MLEs, in this section, we focus on deriving constrained MLEs given the assumption that the odds ratios are homogeneous across strata. With that being said, we now derive the constrained MLEs of *π*_*ij*_, *ρ*_*j*_ and *θ*, denoted by ${\tilde{\pi }}_{ij}$, ${\tilde{\rho }}_{j}$ and $\tilde{\theta }$ under the null hypothesis of *θ*_1_ = … = *θ*_*J*_ = *θ*. Here, *θ* is not fixed and could take on a range of reasonable values.

We transform the homogeneity constraint into $\pi_{2j}=\frac{\pi_{1j}}{\pi_{1j}\left(1-\theta \right)+\theta }$ for any *j* ∈ {1, …, *J*}. Thus, the log-likelihood function under the homogeneity constraint in the *j*^*th*^ stratum can be further expressed as:

$$\begin{array}{ll} l_{j|\theta }(\pi_{1j},\rho_{j},\theta ) & =n_{0+j}\;\text{ln}(\pi_{1j})+n_{1+j}\;\text{ln}(1-\pi_{1j})-n_{+2j}\;\text{ln}((1-\pi_{1j})\theta +\pi_{1j})\\ & +m_{01j}\;\text{ln}(\rho_{j}\pi_{1j}-\pi_{1j}+1)+m_{02j}\;\text{ln}(\rho_{j}\pi_{1j}-\pi_{1j}\theta +\theta )+m_{0+j}\;\text{ln}(1-\pi_{1j})\\ & -m_{1+j}\;\text{ln}((\rho_{j}-1)\pi_{1j}(1-\pi_{1j}))\\ & +m_{21j}\;\text{ln}(\rho_{j}(1-\pi_{1j})+\pi_{1j})+m_{22j}\;\text{ln}(\rho_{j}\theta (1-\pi_{1j})+\pi_{1j})+m_{2+j}\;\text{ln}(\pi_{1j})\\ & -m_{+2j}\;\text{ln}{\left(\pi_{1j}(1-\theta )+\theta \right)}^{2}, \end{array}$$

and the log-likelihood function for all strata is:

$$l_{H_{0}|\theta }\left(\pi_{1},\rho ,\theta \right)=\sum_{j=1}^{J}l_{j|\theta }\left(\pi_{1j},\rho_{j},\theta \right).$$

We adapt a Newton Raphson method to update constrained MLE of *θ*, denoted by $\tilde{\theta }$, and a Fisher Scoring method to update constrained MLEs of *π*_1*j*_ and *ρ*_*j*_ simultaneously, denoted by ${\tilde{\pi }}_{1j}$ and ${\tilde{\rho }}_{j}$. The ensuing “two-steps” algorithm 2 is designed to reduce the dimension of the information matrix, which, facilitates fast and robust convergence during the computation process.

**Algorithm 2 Newton Raphson & Fisher Scoring method for** ${\tilde{\pi }}_{ij}$ , ${\tilde{\rho }}_{j}$ **and** $\tilde{\theta }$

1. Set initial values of $\pi_{1j}^{\left(0\right)}=\frac{1}{J}\sum_{j=1}^{J}{\hat{\pi }}_{1j}$, $\rho_{j}^{\left(0\right)}=\frac{1}{J}\sum_{j=1}^{J}{\hat{\rho }}_{j}$, and $\theta^{\left(0\right)}=\frac{1}{J}\sum_{j=1}^{J}{\hat{\theta }}_{j}$.

2. For *t* = (0, 1, …) until convergence:

 (a) For *k* = (0, 1, …) until convergence, update *θ*^(*k*+1)^ based on

$$\theta^{\left(k+1\right)}=\theta^{\left(k\right)}-{\left(\frac{\partial^{2}l_{H_{0}|\theta }}{\partial \theta^{2}}\left(\pi_{1j}^{\left(t\right)},\rho_{j}^{\left(t\right)},\theta^{\left(k\right)}\right)\right)}^{-1}\frac{\partial l_{H_{0}|\theta }}{\partial \theta }\left(\pi_{1j}^{\left(t\right)},\rho_{j}^{\left(t\right)},\theta^{\left(k\right)}\right).$$

 (b) Update $\pi_{1j}^{\left(t+1\right)}$ and $\rho_{j}^{\left(t+1\right)}$ simultaneously based on

$$(\begin{array}{c} \pi_{1j}^{\left(t+1\right)}\\ \rho_{j}^{\left(t+1\right)} \end{array})=(\begin{array}{c} \pi_{1j}^{\left(t\right)}\\ \rho_{j}^{\left(t\right)} \end{array})+I^{-1}\left(\pi_{1j}^{\left(t\right)},\rho_{j}^{\left(t\right)},\theta^{\left(k\right)}\right)(\begin{array}{c} \frac{\partial l_{j|\theta }}{\partial \pi_{1j}}\left(\pi_{1j}^{\left(t\right)},\rho_{j}^{\left(t\right)},\theta^{\left(k\right)}\right)\\ \frac{\partial l_{j|\theta }}{\partial \rho_{j}}\left(\pi_{1j}^{\left(t\right)},\rho_{j}^{\left(t\right)},\theta^{\left(k\right)}\right) \end{array}),$$

 where

$$I^{-1}\left(\pi_{1j}^{\left(t\right)},\rho_{j}^{\left(t\right)},\theta^{\left(k\right)}\right)=E(\begin{array}{cc} -\frac{\partial^{2}l_{j|\theta }}{\partial \pi_{1j}^{2}} & -\frac{\partial^{2}l_{j|\theta }}{\partial \pi_{1j}\partial \rho_{j}}\\ -\frac{\partial^{2}l_{j|\theta }}{\partial \pi_{1j}\partial \rho_{j}} & -\frac{\partial^{2}l_{j|\theta }}{\partial \rho_{j}^{2}} \end{array}).$$

3. Calculate ${\tilde{\pi }}_{2j}=\frac{{\tilde{\pi }}_{1j}}{{\tilde{\pi }}_{1j}\left(1-\tilde{\theta }\right)+\tilde{\theta }}$.

### Testing methods

In this section, we propose three large-sample testing methods: the likelihood ratio test, the score test and the Wald test, to assess the homogeneity of odds ratios across strata with null hypothesis H_0_: *θ*_1_ = *θ*_2_ = … = *θ*_*J*_ ≜ *θ* versus the alternative hypothesis *H*_*a*_: *θ*_*r*_ ≠ *θ*_*s*_, for at least one pair (*r*, *s*) ∈ {1, …, *J*} and *r* ≠ *s*. Our focus is on the existence of homogeneity, and we derive the test statistics and testing procedures correspondingly as follows.

#### Likelihood ratio test

The likelihood ratio test statistics is constructed by:

$$T_{LR}=2\sum_{j=1}^{J}[l_{j}\left({\hat{\pi }}_{ij},{\hat{\rho }}_{j},{\hat{\theta }}_{j}\right)-l_{j|\theta }\left({\tilde{\pi }}_{1j},{\tilde{\rho }}_{j},\tilde{\theta }\right)].$$

It is noteworthy that under the null hypothesis, *T*_*LR*_ is asymptotically distributed as a chi-square distribution with *J* − 1 degrees of freedom. The following score test and the Wald test have the same asymptotic distributions as the likelihood ratio test.

#### Score test

Let $U_{j}=\left(\frac{\partial l_{j}}{\partial \theta_{j}},\frac{\partial l_{j}}{\partial \pi_{1j}},\frac{\partial l_{j}}{\partial \rho_{j}}\right)$, let ***α***_*j*_ = (*θ*_*j*_, *π*_1*j*_, *ρ*_*j*_)^*T*^. To construct score test in the homogeneity setting, *θ*_*j*_ is the parameter of interest, while *π*_1*j*_ and *ρ*_*j*_ are nuisance parameters under the null hypothesis. Thus, the score function can be simplified as $U_{j}=\left(\frac{\partial l_{j}}{\partial \theta_{j}},0,0\right)$, and the score test statistics can be constructed by:

$$T_{SC}=\sum_{j=1}^{J}U_{j}I_{j}^{-1}\left({\tilde{\alpha }}_{j}\right)U_{j}^{T}{|}_{{\tilde{\alpha }}_{j}=\left(\tilde{\theta },{\tilde{\pi }}_{1j},{\tilde{\rho }}_{j}\right)}.$$

More steps can be found in S1 Appendix.

#### Wald test

As stated by [22], the skewed sampling distribution of odds ratio in logistic regression often results in extreme value, particularly when applied to smaller samples. As the distribution of the log-transformed odds ratio converges more rapidly to a normal distribution [23], we apply a log-transformation via delta method to enhance the robustness of the Wald test. Let ***β*** = (*π*_11_, *π*_21_, *ρ*_1_, …, *π*_1*J*_, *π*_2*J*_, *ρ*_*J*_)^*T*^. We transform the null hypothesis *θ*_1_ = … = *θ*_*J*_ = *θ* under homogeneity into a matrix form of ***Cδ*** = **0**, where

$$\begin{array}{c} C\equiv (\begin{array}{cccccccccccccccccc} 1 & -1 & 0 & -1 & 1 & 0 & 0 & 0 & 0 & \cdots & \cdots & \cdots & 0 & 0 & 0 & 0 & 0 & 0\\ 0 & 0 & 0 & 1 & -1 & 0 & -1 & 1 & 0 & \cdots & \cdots & \cdots & 0 & 0 & 0 & 0 & 0 & 0\\ \vdots & \vdots & \vdots & \vdots & \vdots & \vdots & \vdots & \vdots & \vdots & \ddots & \ddots & \ddots & \vdots & \vdots & \vdots & \vdots & \vdots & \vdots \\ 0 & 0 & 0 & 0 & 0 & 0 & 0 & 0 & 0 & \cdots & \cdots & \cdots & 1 & -1 & 0 & -1 & 1 & 0 \end{array}{)}_{(J-1)\times 3J}, \end{array}$$

and $\delta \equiv {\left(\text{log}\left(\frac{\pi_{11}}{1-\pi_{11}}\right),\text{log}\left(\frac{\pi_{21}}{1-\pi_{21}}\right),\text{log}\left(\rho_{1}\right),\ldots ,\text{log}\left(\frac{\pi_{1J}}{1-\pi_{1J}}\right),\text{log}\left(\frac{\pi_{2J}}{1-\pi_{2J}}\right),\text{log}\left(\rho_{J}\right)\right)}^{T}$.

Note that the asymptotic distribution of maximum likelihood estimator $\hat{\beta }$ is $\sqrt{n}\left(\hat{\beta }-\beta \right)\overset{d}{\to }N\left(0,I_{\beta }^{-1}\right),$ where ***I***_***β***_ is the information matrix for ***β***. According to delta method, we have $\sqrt{n}\left(\hat{\delta }-\delta \right)\overset{d}{\to }N\left(0,I_{\delta }^{-1}\right),$ where $I_{\delta }^{-1}=\Delta_{g}I_{\beta }^{-1}\Delta_{g}^{T}$, and Δ_*g*_ is of a block diagonal structure diag(*g*_1_, …, *g*_*J*_) with

$$\begin{array}{c} g_{j}=(\begin{array}{ccc} \frac{1}{\pi_{1j}\left(1-\pi_{1j}\right)} & 0 & 0\\ 0 & \frac{1}{\pi_{2j}\left(1-\pi_{2j}\right)} & 0\\ 0 & 0 & \frac{1}{\rho_{j}} \end{array}),j\in \left\{1,\ldots ,J\right\}. \end{array}$$

Thus, the Wald test statistics is constructed by:

$$\begin{array}{cc} T_{W} & ={\left(C\hat{\delta }\right)}^{T}Var^{-1}\left(C\hat{\delta }\right)\left(C\hat{\delta }\right)\\ & =\left({\hat{\delta }}^{T}C^{T}\right){\left(C\Delta_{g}I_{\hat{\beta }}^{-1}\Delta_{g}^{T}C^{T}\right)}^{-1}\left(C\hat{\delta }\right){|}_{\hat{\beta }=\left({\hat{\pi }}_{11},{\hat{\pi }}_{21},{\hat{\rho }}_{1},\ldots ,{\hat{\pi }}_{1J},{\hat{\pi }}_{2J},{\hat{\rho }}_{J}\right)}. \end{array}$$

More steps can be found in S1 Appendix.

## Monte Carlo simulation studies

In this section, we evaluate the performance of the proposed testing methods by empirical type I error rates and power. For type I error, Monte Carlo simulations are conducted with fixed and random data generating settings to assess the robustness of our approach. In the case of fixed settings, we consider specific values for the event rate in the treatment group (*π*_1*j*_ ∈ {0.2, 0.3, 0.4}), the intraclass correlation (*ρ*_*j*_ ∈ {0.4, 0.5, 0.6}), and the odds ratio (*θ* ∈ {1.0, 1.2, 1.5, 2.0}), to generate 50, 000 replicates for each configuration, encompassing a diverse range of sample sizes and strata. For random settings, 1, 000 sets of (*π*_1*j*_, *ρ*_*j*_, *θ*) are randomly chosen from *π*_1*j*_ ∈ (0, 1), *ρ*_*j*_ ∈ (0, 1), and *θ* ∈ [1, 4], to emulate real-world scenarios to the greatest extent possible. We calculate the empirical type I error rate as the number of rejections under the null hypothesis divided by the number of MC replicates.

For power assessment, we compare the empirical power with same parameter settings for *π*_1*j*_ and *ρ*_*j*_ aforementioned. The selection of *θ*_*j*_s in the alternative hypotheses are properly specified across different strata, as outlined in Table 2. The empirical power is then computed as the number of rejections of the null hypothesis divided by the number of MC replicates given that the alternative hypothesis holds true.

**Table 2:** The settings of *θ*_*j*_, *j* ∈ {1, …, *J*} for data generation under the alternative hypotheses for power calculation.

Scenarios	Number of Strata		
	J = 2	J = 4	J = 8
	(*θ*_1_, *θ*_2_)	(*θ*_1_, *θ*_2_, *θ*_3_, *θ*_4_)	(*θ*_1_, *θ*_2_, *θ*_3_, *θ*_4_, *θ*_5_, *θ*_6_, *θ*_7_, *θ*_8_)
*H*_*a*1_	(1.0, 1.5)	(1.0, 1.2, 1.5, 2.0)	(1.0, 1.2, 1.5, 2.0, 1.0, 1.2, 1.5, 2.0)
*H*_*a*2_	(1.0, 2.0)	(1.0, 2.0, 1.0, 2.0)	(1.0, 2.0, 1.0, 2.0, 1.0, 2.0, 1.0, 2.0)
*H*_*a*3_	(1.0, 3.0)	(2.0, 4.0, 2.0, 4.0)	(2.0, 4.0, 2.0, 4.0, 2.0, 4.0, 2.0, 4.0)
*H*_*a*4_	(1.0, 4.0)	(1.0, 2.0, 3.0, 4.0)	(1.0, 2.0, 3.0, 4.0, 1.0, 2.0, 3.0, 4.0)

### Type I error

The performance of empirical size of the three proposed tests are presented in Tables 3–5. For small samples, as indicated by Table 3, the likelihood ratio test and the score test are conservative when the number of strata is small. Conversely, as shown in Table 5, the Wald test becomes conservative as the number of strata increases, while the likelihood ratio test and the score test have better performance. We observe that smaller values of *π*_1*j*_ and larger values of *θ* tend to induce more conservative behaviors, while the values of the intraclass correlation *ρ*_*j*_ has minimal impact on the empirical size. Overall, all three proposed tests exhibit empirical type I error rates at the nominal level under all scenarios in large sample sizes, and our simulation results indicate tendencies toward conservative behaviors in small sample sizes [12].

**Table 3:** The empirical type I error rates for 2 strata based on 50, 000 replicates at the 0.05 significance level for testing *H*_0_: *θ*_*j*_ = *θ*, ∀*j* ∈ {1, …, *J*} versus *H*_*a*_: *θ*_*j*_ ≠ *θ*, ∃*j* ∈ {1, …, *J*}.

*m*_+*ij*_ = *n*_+*ij*_	*π*_1*j*_	*ρ*_*j*_	*H*_0*A*_: *θ* = 1			*H*_0*B*_: *θ* = 1.2			*H*_0*C*_: *θ* = 1.5			*H*_0*D*_: *θ* = 2		
			*T*_*LR*_	*T*_*SC*_	*T*_*W*_	*T*_*LR*_	*T*_*SC*_	*T*_*W*_	*T*_*LR*_	*T*_*SC*_	*T*_*W*_	*T*_*LR*_	*T*_*SC*_	*T*_*W*_
30	0.2	0.4	5.21	4.21	4.93	4.14	4.04	4.74	**3.54**	**3.53**	4.86	**3.41**	**3.25**	4.53
		0.5	4.31	4.48	4.81	4.03	**3.98**	4.72	**3.59**	**3.54**	4.93	**3.42**	**3.27**	4.55
		0.6	4.15	4.38	4.96	**3.97**	**3.93**	4.82	**3.49**	**3.30**	4.79	**3.29**	**3.17**	4.68
	0.3	0.4	5.07	5.07	4.99	4.93	4.82	5.06	4.69	4.59	4.98	4.10	4.00	4.78
		0.5	5.11	5.10	5.06	4.83	4.88	5.20	4.76	4.60	5.14	**3.91**	**3.80**	4.95
		0.6	5.10	4.91	5.05	4.97	4.79	5.19	4.51	4.50	5.16	**3.82**	**3.67**	5.04
	0.4	0.4	5.25	5.08	5.02	5.01	4.97	5.15	5.29	5.06	4.94	4.83	4.56	4.93
		0.5	5.04	5.01	5.18	5.27	5.05	5.03	5.06	5.04	5.03	4.88	4.74	5.25
		0.6	5.12	5.14	4.98	5.14	4.93	4.99	4.94	4.92	5.13	4.58	4.46	5.23
50	0.2	0.4	4.98	4.97	4.96	5.07	4.90	5.07	4.62	4.62	5.17	**3.93**	**3.76**	4.90
		0.5	5.15	5.08	4.85	5.06	4.82	5.02	4.58	4.50	5.10	**3.94**	**3.86**	4.96
		0.6	5.16	5.00	4.97	4.96	4.92	4.74	4.45	4.33	5.09	4.04	**3.79**	4.93
	0.3	0.4	5.15	5.36	5.25	5.21	5.03	5.14	5.17	4.94	5.15	5.00	5.02	4.94
		0.5	5.21	5.09	5.14	5.06	5.20	5.24	5.11	5.05	5.06	4.86	4.68	5.11
		0.6	4.99	5.26	4.90	5.12	5.13	5.06	5.10	5.04	4.97	4.84	4.87	5.01
	0.4	0.4	5.24	4.96	5.15	4.96	5.08	5.14	5.04	5.06	5.14	5.03	5.06	5.12
		0.5	5.15	5.14	4.91	5.19	4.98	5.12	5.23	5.15	4.98	5.35	5.03	5.05
		0.6	5.01	5.07	5.10	5.08	4.99	5.10	4.95	5.18	5.10	5.01	5.06	5.04
100	0.2	0.4	5.25	5.14	4.90	5.11	5.09	5.15	5.06	4.94	5.07	5.04	4.99	5.00
		0.5	5.11	5.08	5.01	5.11	5.06	4.94	5.02	5.05	5.13	4.98	4.96	4.84
		0.6	5.04	4.99	5.01	5.02	5.05	4.99	5.11	5.06	5.04	4.97	5.04	5.02
	0.3	0.4	5.16	4.96	5.20	5.09	5.00	5.10	5.08	5.25	5.24	5.14	4.95	5.11
		0.5	4.97	5.12	5.04	5.00	5.12	5.09	5.11	5.21	5.02	5.10	5.01	5.15
		0.6	5.09	5.10	4.76	4.98	5.15	4.90	5.10	4.90	5.04	5.06	4.93	5.16
	0.4	0.4	5.11	5.11	4.89	5.18	5.06	4.99	5.14	5.10	4.89	5.15	5.00	5.00
		0.5	5.10	4.98	4.99	5.15	5.00	5.09	5.01	5.15	5.09	5.06	5.00	5.04
		0.6	4.92	4.88	5.03	5.31	5.09	4.99	5.00	5.11	5.04	5.13	4.89	5.29

Note: the conservative empirical sizes (less than 4.00) are shown in bold.

**Table 4:** The empirical type I error rates for 4 strata based on 50, 000 replicates at the 0.05 significance level for testing *H*_0_: *θ*_*j*_ = *θ*, ∀*j* ∈ {1, …, *J*} versus *H*_*a*_: *θ*_*j*_ ≠ *θ*, ∃*j* ∈ {1, …, *J*}.

*m*_+*ij*_ = *n*_+*ij*_	*π*_1*j*_	*ρ*_*j*_	*H*_0*A*_: *θ* = 1			*H*_0*B*_: *θ* = 1.2			*H*_0*C*_: *θ* = 1.5			*H*_0*D*_: *θ* = 2		
			*T*_*LR*_	*T*_*SC*_	*T*_*W*_	*T*_*LR*_	*T*_*SC*_	*T*_*W*_	*T*_*LR*_	*T*_*SC*_	*T*_*W*_	*T*_*LR*_	*T*_*SC*_	*T*_*W*_
30	0.2	0.4	4.44	4.03	4.38	4.47	**3.88**	4.29	4.10	**3.84**	4.17	4.27	4.03	**3.69**
		0.5	4.36	4.22	4.41	4.19	**3.97**	4.27	4.16	**3.99**	4.12	4.22	**3.98**	**3.91**
		0.6	4.37	4.26	4.35	4.33	4.14	4.28	4.10	4.26	4.12	4.33	**3.97**	**3.77**
	0.3	0.4	4.86	4.63	4.87	4.78	4.50	4.87	4.62	4.21	4.87	4.27	4.18	4.72
		0.5	4.84	4.60	5.11	4.76	4.61	4.93	4.69	4.51	4.88	4.39	4.26	4.72
		0.6	4.85	4.68	4.83	4.70	4.56	4.89	4.45	4.41	4.95	4.43	4.43	4.65
	0.4	0.4	5.19	4.88	5.02	4.96	4.90	4.99	4.75	4.69	5.15	4.67	4.58	4.94
		0.5	5.00	4.84	5.21	4.68	4.83	5.08	4.85	4.78	5.20	4.71	4.43	4.93
		0.6	4.92	4.91	4.88	4.85	4.79	4.98	4.74	4.64	5.07	4.51	4.50	4.85
50	0.2	0.4	4.83	4.68	4.88	4.84	4.58	4.82	4.72	4.41	4.63	4.29	4.29	4.55
		0.5	4.88	4.73	4.80	4.64	4.51	4.87	4.71	4.50	4.70	4.37	4.27	4.59
		0.6	4.82	4.92	4.85	4.56	4.48	4.71	4.48	4.41	4.57	4.53	4.17	4.36
	0.3	0.4	5.14	4.80	4.99	5.27	4.91	4.85	5.00	4.75	4.97	4.83	4.69	4.77
		0.5	5.14	4.98	5.01	4.91	5.04	4.96	4.77	4.64	4.80	4.70	4.60	4.82
		0.6	5.00	5.04	5.04	4.97	5.09	5.03	4.90	4.56	4.95	4.50	4.59	4.94
	0.4	0.4	5.38	5.11	5.03	5.23	5.14	5.00	5.17	5.06	5.15	5.05	4.71	4.74
		0.5	5.08	5.08	4.99	5.24	5.04	5.12	4.92	4.99	5.06	5.08	4.83	4.78
		0.6	5.18	5.03	5.01	5.19	5.14	5.01	5.06	5.00	5.04	4.98	4.93	5.08
100	0.2	0.4	5.08	5.06	5.11	5.06	5.05	4.86	4.98	4.96	4.91	4.84	4.67	4.90
		0.5	5.14	5.09	5.17	5.08	4.77	4.93	5.00	4.90	5.02	4.95	4.76	4.90
		0.6	5.14	4.99	4.95	5.08	4.98	4.88	4.87	4.83	4.92	4.76	4.54	4.98
	0.3	0.4	5.24	5.01	5.11	5.00	5.10	4.94	5.20	5.12	5.10	5.01	4.94	4.98
		0.5	4.90	4.89	4.97	5.12	5.12	4.95	5.05	4.93	4.84	5.08	5.05	4.99
		0.6	5.21	5.12	4.98	5.06	5.04	5.04	5.09	5.07	5.19	5.19	4.91	4.85
	0.4	0.4	5.08	5.07	5.13	4.93	4.96	5.09	5.09	4.98	5.28	5.11	5.04	5.13
		0.5	5.17	5.10	5.04	5.10	5.08	4.91	5.00	5.14	5.00	5.16	5.08	4.98
		0.6	4.89	5.03	4.75	4.99	5.27	5.06	5.27	4.96	5.11	5.05	5.01	4.86

Note: the conservative empirical sizes (less than 4.00) are shown in bold.

**Table 5:** The empirical type I error rates for 8 strata based on 50, 000 replicates at the 0.05 significance level for testing *H*_0_: *θ*_*j*_ = *θ*, ∀*j* ∈ {1, …, *J*} versus *H*_*a*_: *θ*_*j*_ ≠ *θ*, ∃*j* ∈ {1, …, *J*}.

*m*_+*ij*_ = *n*_+*ij*_	*π*_1*j*_	*ρ*_*j*_	*H*_0*A*_: *θ* = 1			*H*_0*B*_: *θ* = 1.2			*H*_0*C*_: *θ* = 1.5			*H*_0*D*_: *θ* = 2		
			*T*_*LR*_	*T*_*SC*_	*T*_*W*_	*T*_*LR*_	*T*_*SC*_	*T*_*W*_	*T*_*LR*_	*T*_*SC*_	*T*_*W*_	*T*_*LR*_	*T*_*SC*_	*T*_*W*_
30	0.2	0.4	4.68	4.12	**3.96**	4.34	4.22	4.16	4.44	4.00	**3.78**	4.55	**3.94**	**3.24**
		0.5	4.62	4.56	**3.99**	4.66	4.23	**3.84**	4.60	4.11	**3.75**	4.53	4.20	**3.21**
		0.6	4.22	4.15	**3.91**	4.14	4.21	**3.76**	4.28	4.09	**3.58**	4.31	4.04	**3.22**
	0.3	0.4	4.92	4.72	4.78	5.08	4.70	4.73	4.88	4.50	4.70	5.01	4.48	4.50
		0.5	5.08	4.80	4.79	4.98	4.78	4.85	5.20	4.61	4.63	4.89	4.42	4.35
		0.6	5.05	4.86	4.69	5.01	4.80	4.77	4.86	4.77	4.62	4.85	4.43	4.40
	0.4	0.4	5.10	4.81	4.99	5.08	4.90	5.01	5.07	4.75	4.68	4.89	4.43	4.77
		0.5	5.11	4.89	4.93	5.27	4.96	4.98	5.17	4.76	4.93	5.11	4.77	4.96
		0.6	4.88	5.04	5.01	5.05	4.85	4.99	4.92	4.77	4.92	4.89	4.75	4.83
50	0.2	0.4	4.96	4.95	4.64	5.08	4.66	4.56	4.84	4.45	4.45	4.92	4.43	4.26
		0.5	4.91	4.75	4.57	5.00	4.71	4.61	5.00	4.52	4.48	4.81	4.56	4.15
		0.6	4.89	4.85	4.56	4.85	4.84	4.47	4.93	4.54	4.15	4.68	4.49	4.09
	0.3	0.4	5.24	4.92	4.96	5.13	4.92	4.97	5.02	5.03	4.87	4.99	4.86	4.69
		0.5	5.26	4.99	5.04	5.20	4.78	4.87	5.09	4.86	4.79	4.98	4.90	4.85
		0.6	5.30	5.03	4.73	5.07	5.08	4.80	5.00	4.81	4.53	4.85	4.75	4.61
	0.4	0.4	5.25	5.23	5.20	5.26	4.95	5.12	5.09	5.00	4.89	5.24	4.99	4.95
		0.5	5.27	5.02	5.03	5.26	4.98	5.09	5.30	4.93	5.05	5.33	4.77	4.80
		0.6	5.17	4.93	5.00	5.09	5.17	4.87	5.06	4.97	4.81	4.90	4.97	5.00
100	0.2	0.4	5.14	4.82	4.84	5.03	4.96	4.76	5.15	4.86	4.63	4.96	4.88	4.75
		0.5	5.01	4.98	4.93	5.02	4.86	4.97	5.10	4.89	4.91	4.82	4.82	4.67
		0.6	5.30	4.95	4.80	5.11	4.89	4.69	5.18	4.89	4.57	4.93	4.66	4.58
	0.3	0.4	5.27	5.19	4.96	5.23	5.05	5.16	5.23	4.89	5.12	5.25	4.94	4.77
		0.5	5.08	5.00	5.01	5.18	4.97	4.84	5.31	5.17	4.78	5.04	5.02	5.00
		0.6	5.18	5.02	5.00	5.24	5.11	4.95	5.02	5.03	4.95	5.05	4.98	5.05
	0.4	0.4	5.09	4.94	5.10	4.97	4.90	4.98	5.07	5.01	5.03	5.03	5.17	5.04
		0.5	5.21	5.38	4.87	5.14	5.00	5.04	5.14	5.07	5.04	5.16	5.02	5.17
		0.6	5.10	5.18	4.87	5.10	5.04	5.02	5.26	5.09	5.01	5.08	5.11	4.94

Note: the conservative empirical sizes (less than 4.00) are shown in bold.

**Fig 1:**
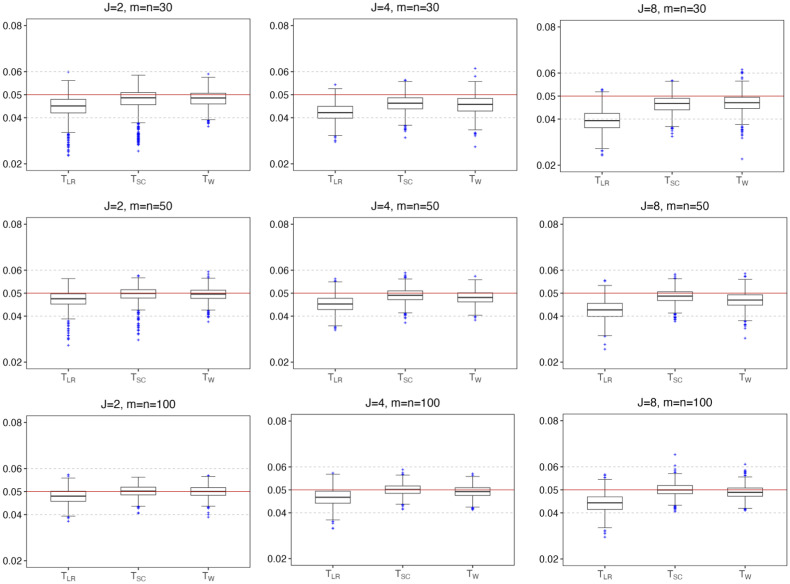
Box plots of the empirical type I error rates for 1, 000 random parameter settings of (*π*, *ρ*, *θ*) considering *J* ∈ {2, 4, 8} and *m* = *n* ∈ {30, 50, 100} (*m* = *m*_+*ij*_, *n* = *n*_+*ij*_). In each setting the empirical type I error rate is calculated based on 10, 000 replicates at 0.05 the significance level.

In addition to the consideration of fixed parameter settings, Fig 1 presents the dispersion of the empirical type I errors based on 1, 000 sets of (*π*_1*j*_, *ρ*_*j*_, *θ*) that are randomly chosen from *π*_1*j*_ ∈ (0, 1), *ρ*_*j*_ ∈ (0, 1), and *θ* ∈ [1, 4]. Within each random set of parameters, 10, 000 MC replicates are generated under the null hypothesis of homogeneity. We observe that the likelihood ratio test is conservative in small and moderate samples. The Wald test tends to become conservative in small samples, particularly as the number of strata increases. The score test performs well in moderate and large sample sizes regardless of the number of strata, thus, is recommended overall.

### Power

The empirical power performance of the three proposed tests is presented in Tables 6–8. It is obvious that the powers increase as the the sample sizes increase. Furthermore, a higher degree of heterogeneity in odds ratios among strata is associated with a greater magnitude of statistical power. In general, the likelihood ratio test and the score test have greater statistical power compared to the Wald test, although their performance tends to be similar as sample sizes become large. In addition, these simulation-based power tables offer insights into the required sample sizes for achieving certain power levels in practical scenarios where the availability or adaptability of relevant sample size formulas may be limited. For example, *m* = *n* = 50 is sufficient to achieve 80% statistical power at the 0.5 significance level in testing *H*_0_: *θ*_1_ = *θ*_2_ = *θ*_3_ = *θ*_4_ versus *H*_*a*_: *θ*_1_ = 1.0, *θ*_2_ = 1.2, *θ*_3_ = 1.5, *θ*_4_ = 2 when there are 4 strata.

**Table 6:** The empirical power for 2 strata based on 50, 000 replicates for testing *H*_0_: *θ*_*j*_ = *θ*, ∀*j* ∈ {1, …, *J*} versus *H*_*a*_: *θ*_*j*_ ≠ *θ*, ∃*j* ∈ {1, …, *J*}.

*m*_+*ij*_ = *n*_+*ij*_	*π*_1*j*_	*ρ*_*j*_	*H*_*a*1_			*H*_*a*2_			*H*_*a*3_			*H*_*a*4_		
			*T*_*LR*_	*T*_*SC*_	*T*_*W*_	*T*_*LR*_	*T*_*SC*_	*T*_*W*_	*T*_*LR*_	*T*_*SC*_	*T*_*W*_	*T*_*LR*_	*T*_*SC*_	*T*_*W*_
30	0.2	0.4	10.43	10.47	10.13	20.70	20.14	19.25	40.39	39.49	38.23	54.67	53.07	51.17
		0.5	10.49	10.10	9.69	20.01	19.57	18.89	39.26	38.06	36.62	53.25	51.98	49.05
		0.6	9.85	10.12	9.76	19.14	19.04	18.41	37.56	36.92	35.49	50.90	50.61	46.91
	0.3	0.4	12.56	11.96	12.15	25.77	25.70	25.69	51.55	51.00	50.80	68.45	67.49	67.23
		0.5	12.05	12.04	11.81	25.56	25.14	24.63	49.96	49.58	49.35	67.58	66.11	65.64
		0.6	12.01	11.94	11.39	24.60	24.11	23.98	48.60	48.30	47.49	65.31	64.62	63.97
	0.4	0.4	13.73	13.33	13.54	29.54	29.35	29.27	58.84	58.28	58.24	76.61	76.46	76.14
		0.5	13.30	13.14	13.06	28.46	28.52	27.97	57.29	56.66	56.92	75.01	74.98	74.69
		0.6	12.95	12.90	12.72	27.75	27.10	27.62	56.19	55.19	55.17	73.59	73.40	72.99
50	0.2	0.4	14.46	14.18	14.05	31.16	30.49	30.01	59.62	58.94	58.67	76.30	75.52	75.09
		0.5	14.13	13.76	13.74	29.61	29.45	29.27	58.45	57.28	57.23	74.82	74.25	73.25
		0.6	13.70	13.66	13.25	29.02	28.94	28.27	56.06	56.12	55.29	73.46	72.72	71.36
	0.3	0.4	17.38	17.17	17.00	39.07	39.17	39.01	73.41	72.89	72.84	88.30	87.87	87.96
		0.5	17.00	16.91	16.61	38.50	38.14	37.81	71.39	71.00	70.86	86.96	86.76	86.91
		0.6	16.42	16.50	16.22	36.91	36.41	36.41	69.94	69.52	69.02	85.66	85.53	85.33
	0.4	0.4	19.16	19.05	19.28	45.05	44.46	44.72	80.21	80.39	80.20	93.33	93.49	93.23
		0.5	18.52	18.77	18.79	43.42	43.12	43.04	78.89	78.65	78.34	92.66	92.36	92.39
		0.6	18.57	18.50	18.34	41.72	42.25	42.14	77.26	77.47	76.91	91.60	91.39	91.39
100	0.2	0.4	23.66	23.63	23.15	53.69	53.75	53.42	87.54	87.43	87.24	96.68	96.55	96.43
		0.5	22.78	23.06	22.57	52.31	52.01	52.16	86.42	86.29	85.74	96.00	95.92	96.02
		0.6	22.04	22.18	22.23	50.86	50.44	49.86	85.06	84.59	84.67	95.52	95.26	95.25
	0.3	0.4	29.68	29.58	29.61	66.35	66.35	66.36	95.17	95.36	95.09	99.37	99.40	99.35
		0.5	29.18	28.47	28.74	64.48	64.46	64.46	94.42	94.36	94.35	99.18	99.15	99.17
		0.6	27.81	27.80	27.86	62.87	62.91	63.13	93.97	93.93	93.60	98.99	98.98	99.03
	0.4	0.4	33.53	32.97	33.24	73.38	73.41	73.04	97.78	97.67	97.85	99.83	99.82	99.83
		0.5	32.46	32.36	32.35	71.39	71.47	71.27	97.38	97.28	97.31	99.78	99.78	99.81
		0.6	31.21	31.82	31.03	69.33	70.01	69.84	96.78	96.97	96.92	99.71	99.68	99.67

**Table 7:** The empirical power for 4 strata based on 50, 000 replicates for testing *H*_0_: *θ*_*j*_ = *θ*, ∀*j* ∈ {1, …, *J*} versus *H*_*a*_: *θ*_*j*_ ≠ *θ*, ∃*j* ∈ {1, …, *J*}.

*m*_+*ij*_ = *n*_+*ij*_	*π*_1*j*_	*ρ*_*j*_	*H*_*a*1_			*H*_*a*2_			*H*_*a*3_			*H*_*a*4_		
			*T*_*LR*_	*T*_*SC*_	*T*_*W*_	*T*_*LR*_	*T*_*SC*_	*T*_*W*_	*T*_*LR*_	*T*_*SC*_	*T*_*W*_	*T*_*LR*_	*T*_*SC*_	*T*_*W*_
30	0.2	0.4	14.49	13.99	12.64	23.53	23.02	21.02	18.19	16.48	11.77	45.77	44.16	39.66
		0.5	14.20	13.74	12.29	23.13	21.74	20.22	17.98	16.21	10.98	44.04	42.26	37.02
		0.6	13.60	13.44	11.96	21.62	21.41	19.38	16.90	15.66	10.53	42.30	41.17	35.52
	0.3	0.4	18.57	18.24	17.56	31.35	30.62	29.92	24.85	23.25	21.67	59.48	58.74	57.17
		0.5	18.46	17.73	16.97	30.52	29.48	28.77	24.48	23.12	20.70	57.84	56.86	55.10
		0.6	17.65	17.40	16.72	29.33	28.55	27.89	23.87	22.22	19.63	56.28	55.06	53.42
	0.4	0.4	21.19	20.97	20.85	36.17	35.43	35.31	30.48	29.56	27.88	68.28	67.75	66.58
		0.5	20.55	20.17	19.85	34.68	34.55	34.19	29.71	28.48	27.55	66.42	65.68	65.11
		0.6	20.22	19.64	19.32	33.40	33.19	33.06	28.7	27.79	26.3	64.22	63.58	62.87
50	0.2	0.4	22.33	21.83	20.56	37.82	37.59	36.21	28.72	26.94	24.46	69.13	68.40	66.57
		0.5	21.95	21.38	19.89	36.95	35.63	34.76	27.73	26.14	23.38	67.60	66.50	64.66
		0.6	21.00	20.77	19.45	35.35	34.80	32.94	26.93	24.92	22.01	65.21	64.33	62.64
	0.3	0.4	29.25	28.67	28.06	49.34	49.37	48.34	39.61	38.79	37.33	82.94	82.90	82.24
		0.5	28.14	27.43	27.14	47.88	47.29	46.74	38.25	37.67	35.72	81.86	81.19	80.42
		0.6	27.16	26.74	26.46	46.31	46.01	45.43	36.86	35.95	34.63	80.07	79.78	79.01
	0.4	0.4	33.54	33.30	32.77	56.93	56.03	56.04	48.60	47.62	47.24	89.75	89.60	89.05
		0.5	32.62	32.10	31.86	55.00	54.67	54.43	46.59	46.14	45.82	88.28	88.31	87.98
		0.6	31.13	30.92	30.73	53.32	53.15	52.56	45.63	44.33	43.39	86.96	86.73	86.60
100	0.2	0.4	41.64	41.43	40.43	68.06	67.69	67.44	52.59	51.99	50.99	95.12	94.93	94.62
		0.5	40.02	39.59	39.19	66.36	65.79	65.67	51.24	49.86	48.75	94.18	94.17	93.73
		0.6	38.53	38.59	37.56	64.62	64.03	63.66	49.80	48.27	46.85	93.31	93.21	92.78
	0.3	0.4	54.10	53.77	53.49	81.40	81.65	81.47	70.16	69.96	69.16	98.96	98.95	99.04
		0.5	52.24	52.21	52.14	80.03	79.77	79.57	68.25	67.46	67.17	98.72	98.63	98.64
		0.6	50.66	50.36	49.85	78.15	78.25	77.87	66.88	66.39	65.48	98.33	98.40	98.44
	0.4	0.4	61.57	61.70	61.01	88.00	87.86	87.92	80.68	80.30	80.08	99.71	99.67	99.69
		0.5	59.95	59.35	58.93	86.56	86.37	86.22	78.55	78.44	78.39	99.59	99.62	99.58
		0.6	58.07	57.75	57.98	84.91	85.23	84.93	77.45	76.99	76.15	99.46	99.44	99.51

**Table 8:** The empirical power for 8 strata based on 50, 000 replicates for testing *H*_0_: *θ*_*j*_ = *θ*, ∀*j* ∈ {1, …, *J*} versus *H*_*a*_: *θ*_*j*_ ≠ *θ*, ∃*j* ∈ {1, …, *J*}.

*m*_+*ij*_ = *n*_+*ij*_	*π*_1*j*_	*ρ*_*j*_	*H*_*a*1_			*H*_*a*2_			*H*_*a*3_			*H*_*a*4_		
			*T*_*LR*_	*T*_*SC*_	*T*_*W*_	*T*_*LR*_	*T*_*SC*_	*T*_*W*_	*T*_*LR*_	*T*_*SC*_	*T*_*W*_	*T*_*LR*_	*T*_*SC*_	*T*_*W*_
30	0.2	0.4	17.98	16.86	14.78	32.85	29.99	27.09	23.11	20.71	13.58	63.44	61.40	55.15
		0.5	17.81	16.61	13.96	30.69	29.45	26.00	22.52	20.11	12.62	60.35	59.62	51.86
		0.6	16.43	16.22	13.20	28.40	28.36	24.81	21.44	19.51	11.61	57.64	57.71	49.65
	0.3	0.4	24.63	23.54	22.38	43.60	42.42	40.96	34.17	31.99	28.53	79.22	78.28	76.61
		0.5	23.73	22.98	21.61	42.28	40.85	39.04	33.15	30.84	26.70	77.70	76.57	74.27
		0.6	23.19	22.11	20.88	40.33	39.54	38.26	31.88	29.69	25.47	75.65	74.72	72.16
	0.4	0.4	28.36	27.73	27.55	50.63	49.81	49.13	42.78	40.62	38.83	87.15	86.51	85.95
		0.5	27.51	26.80	26.29	48.72	48.00	47.47	40.80	39.26	37.47	85.36	85.22	84.55
		0.6	26.49	25.52	25.35	46.96	46.20	45.68	39.04	37.80	35.57	83.81	83.24	82.44
50	0.2	0.4	29.92	28.47	27.25	53.20	52.23	50.35	39.41	37.05	32.44	88.14	87.19	85.83
		0.5	28.37	27.42	26.67	51.48	50.48	48.35	38.05	35.59	30.55	86.55	85.64	83.89
		0.6	27.78	26.85	24.64	49.38	48.55	46.36	36.83	33.77	28.73	84.61	84.24	82.05
	0.3	0.4	39.89	39.31	38.57	68.16	67.58	67.16	55.33	53.60	52.01	96.33	96.27	96.07
		0.5	38.58	37.33	37.12	66.35	65.72	64.66	53.79	51.74	49.81	95.67	95.52	95.16
		0.6	37.16	36.06	35.80	64.57	63.69	63.35	51.98	49.70	48.12	94.82	94.79	94.25
	0.4	0.4	47.34	46.02	46.16	76.16	75.99	76.02	66.92	66.21	65.40	98.78	98.63	98.60
		0.5	45.20	44.63	44.71	75.08	74.49	73.98	64.67	63.81	63.19	98.32	98.15	98.21
		0.6	43.39	42.76	42.04	72.59	72.63	72.19	63.12	61.89	61.00	97.89	97.80	97.77
100	0.2	0.4	58.02	57.27	57.29	87.48	86.81	86.41	72.28	71.08	69.76	99.70	99.65	99.65
		0.5	56.54	55.61	54.24	85.88	85.33	84.94	70.33	69.17	67.09	99.60	99.56	99.53
		0.6	54.11	53.70	52.73	83.97	83.70	83.16	68.55	67.01	65.24	99.46	99.46	99.38
	0.3	0.4	73.47	73.20	72.95	95.66	95.62	95.75	88.69	88.28	87.87	100.0	99.99	99.98
		0.5	71.84	71.20	70.83	95.11	94.71	94.64	87.15	86.69	86.29	99.96	99.98	99.98
		0.6	69.61	69.38	68.92	94.08	94.09	93.93	85.70	85.34	84.72	99.97	99.97	99.94
	0.4	0.4	81.21	81.37	81.04	98.16	98.08	98.21	95.37	94.88	94.93	100.0	100.0	100.0
		0.5	79.73	79.51	79.36	97.63	97.64	97.57	94.29	94.22	94.02	100.0	99.99	100.0
		0.6	77.73	77.63	77.18	97.24	97.22	97.11	93.49	93.37	93.03	100.0	100.0	100.0

## Real data examples

In this section, we aim to demonstrate the utility of proposed three testing methods on two real-world datasets. The first data example emanates from a double-blinded randomized clinical trial for studying the efficacy of drug treatments for acute otitis media with effusion [24]. A cohort of 214 children (with a total of 293 ears) who had undergone bilateral or unilateral tympanocentesis were randomly allocated to either of the two groups: one group received a 14-day regimen of cefaclor treatment, while the other group received a 14-day regimen of amoxicillin treatment. Following the completion of the 14-day treatment regimen, 203 children exhibited therapeutic success with the specific counts of effusion-free ears documented in Table 9. The odds ratio is defined as the odds of being cured (effusion-free) in the cefaclor treatment group divided by the odds of being cured in the amoxicillin treatment group. It is of interest to study if the treatment effects of cefaclor versus amoxicillin measured by the odds ratios remain homogeneous across different age strata.

**Table 9:** Frenquencies of individuals with bilateral or unilateral cured (effusion-free) ears at the end of the 14-day course of either cefaclor or amoxicillin treatment under different age at the entry.

Age at the entry	Treatment	No. of effusion-free ears at the end				
		Bilateral at the entry			Unilateral at the entry	
		0	1	2	0	1
< 2 years	Cefaclor	8	2	8	3	9
	Amoxicillin	11	2	2	2	10
2–5 years	Cefaclor	6	6	10	24	7
	Amoxicillin	3	1	5	14	22
≥ 6 years	Cefaclor	0	1	3	11	8
	Amoxicillin	1	0	6	11	7

Table 10 provides the unconstrained and constrained MLEs for the cured rates, the intraclass correlation and odds ratios within both treatment groups. Table 11 presents the corresponding test statistics and the associated p-values based on the three proposed tests. We notice that the test statistics are similar and all fall below the critical value of $\chi_{2,0.95}^{2}=5.9915$, indicating that we have no sufficient evidence to reject the null hypothesis that the odds ratios are homogeneous across different age strata, given the significance level of 5%.

**Table 10:** MLEs under no constaint and MLEs under the constraint of homogeneity calculated based on data.

Age at the entry	Constrained MLEs				Unconstrained MLEs			
	${\tilde{\pi }}_{1j}^{a}$	${\tilde{\pi }}_{2j}^{b}$	${\tilde{\rho }}_{j}$	$\tilde{\theta }$	${\hat{\pi }}_{1j}^{a}$	${\hat{\pi }}_{2j}^{b}$	${\hat{\rho }}_{j}$	$\hat{\theta }$
< 2 years	0.4972	0.5687	0.7677	0.7500	0.5929	0.4616	0.7557	1.6983
2–5 years	0.4627	0.5345	0.5520	0.7500	0.4011	0.6130	0.5587	0.4228
≥ 6 years	0.4820	0.5537	0.8101	0.7500	0.5062	0.5299	0.8176	0.9097

^*a*^ the cured rate in the cefaclor group.

^*b*^ the cured rate in the amoxicillin group.

**Table 11:** Test statistics and p-values calculated based on the three proposed tests.

	*T*_*LR*_	*T*_*SC*_	*T*_*W*_
Statistics	4.7324	4.6925	4.6943
p-value	0.0938	0.0957	0.0956

The second data example stems from an observational study conducted in 2023 at the First Affiliated Hospital of Xiamen University [25]. A total of 60 subjects diagnosed with myopia were recruited to receive Orthokeratology (Ortho-k), a non-surgical vision correction technique by using overnight specialized contact lenses for reshaping corneal. The choice of wearing the lenses on both eyes or a single eye is determined by the preference of the individual patients. There are two lenses treatment options [26]: corneal refractive therapy (CRT) lenses and vision shaping treatment (VST) lenses. In this study, the treatment success or improvement is determined by the criterion of axial length growth being less than 0.3 mm according to [27]. The odds ratio is defined as the odds of being myopia improved in the VST treatment group divided by the odds of being myopia improved in the CRT treatment group. It is of interest to compare the efficacy of the two treatments across different sex strata. The number of myopia improved eyes is summarized in Table 12.

**Table 12:** Frequencies of individuals with bilateral or unilateral myopia improved eyes by receiving either VST or CRT treatment.

Sex	Treatment	No. of myopia improved eyes				
		Bilateral lenses			Unilateral lenses	
		0	1	2	0	1
Female	VST	9	3	7	2	1
	CRT	7	0	0	0	0
Male	VST	11	4	3	1	2
	CRT	6	2	2	0	0

Table 13 provides the unconstrained and constrained MLEs for the myopia improved rates, the intraclass correlation and odds ratios within both treatment groups. The test statistics and p-values for the three proposed tests are presented in Table 14. It is noteworthy that the likelihood ratio test is conservative, aligning with the results from our simulation study when the sample size is small. In this data example, we have sufficient evidence to reject the null hypothesis that the odds ratios are homogeneous across sex strata.

**Table 13:** Constrained and unconstrained MLEs calculated based on data.

Sex	Constrained MLEs				Unconstrained MLEs			
	${\tilde{\pi }}_{1j}^{a}$	${\tilde{\pi }}_{2j}^{b}$	${\tilde{\rho }}_{j}$	$\tilde{\theta }$	${\hat{\pi }}_{1j}^{a}$	${\hat{\pi }}_{2j}^{b}$	${\hat{\rho }}_{j}$	$\hat{\theta }$
Female	0.3735	0.1807	0.7123	2.7039	0.4340	0.0032	0.6801	236.4251
Male	0.3784	0.1838	0.4942	2.7039	0.3215	0.2982	0.4859	1.1154

^*a*^ the improved rate in the VST group.

^*b*^ the improved rate in the CRT group.

**Table 14:** Frequencies of the individuals within the *j*^*th*^ stratum under stratified unilateral and bilateral combined design.

	*T*_*LR*_	*T*_*SC*_	*T*_*W*_
Statistics	5.1120	3.8689	4.3991
p-value	0.0238	0.0492	0.0359

## Discussion

This article proposed three large-sample tests, the likelihood ratio test, the score test, and the Wald test, designed to assess the homogeneity of odds ratios across multiple strata when data are pooled either bilaterally or unilaterally. Simulation results indicate that all three tests demonstrate satisfactory performance in terms of empirical type I error rates when applied to large sample sizes. For small sample sizes, the likelihood ratio test is conservative with small number of strata, while the Wald test becomes conservative with an increasing number of strata. In light of these findings, the score test is recommended for its capacity to maintain a robust control over type I error and power. We illustrated the use of the three proposed tests with a dataset on acute otitis media and another dataset on myopia eyes. Since homogeneity tests are commonly utilized to compare the treatment effects across various subgroups, our proposed tests possess the potential for application in a wide range of clinical scenarios to facilitate informed decision-making.

Our investigation centers on the setting of stratified randomization with correlated combined data which are collected either unilaterally or bilaterally from paired organs. This focus is driven by three key considerations: (i) It is of interest to compare the treatment effects across different strata to under how the treatment works for various patient populations. (ii) Unilateral data arise in clinical practice when the collection of complete bilateral data is challenging. It is critical to develop methodologies that are capable of handling both types of data. (iii) A significant portion of studies tend to analyze paired organs in isolation rather than treating the human as a unified entity with bilateral observations from paired organs. Ignoring such correlation will result in an inflated type I error. Our results indicate that the proposed tests display no tendencies towards liberal behaviors, which demonstrates an effective control over correlation-related variations.

In this work we have proposed homogeneity tests for large samples. In cases where the homogeneity is upheld, we may proceed to test the specific value of the common treatment effect. Confidence intervals can be constructed to make the data interpretation more convincing beyond relying sorely on p-values. In addition, exact methods warrant future research as an alternative to the asymptotic methods, particularly in situations where the latter may work poorly for controlling type I error when dealing with relatively small and sparse data.

## Supporting information

S1 Appendix (PDF)
