# Supplementary material for: Testing the homogeneity of odds ratio across strata for combined bilateral and unilateral data
Source: PLoS One. 2024 Jul 18;19(7):e0307276. doi: 10.1371/journal.pone.0307276 (PMC11257320; doi:10.1371/journal.pone.0307276)
Supplement: S1 Appendix — (PDF) [file pone.0307276.s001.pdf]

Appendix

Score Test

Information Matrix Derivation

Consider the log likelihood function within a stratum under the constraint of homogeneity by substituting $\pi_{2j} = \frac{\pi_{1j}}{\pi_{1j}(1-\theta)+\theta}$, then we have:

$$
\begin{aligned}
l_{j|\theta}(\pi_{1j}, \rho_j, \theta) =& n_{0+j} \ln(\pi_{1j}) + n_{1+j} \ln(1-\pi_{1j}) - n_{+2j} \ln((1-\pi_{1j})\theta + \pi_{1j}) \\
&+ m_{01j} \ln(\rho_j \pi_{1j} - \pi_{1j} + 1) + m_{02j} \ln(\rho_j \pi_{1j} - \pi_{1j}\theta + \theta) + m_{0+j} \ln(1-\pi_{1j}) \\
&- m_{1+j} \ln((\rho_j - 1)\pi_{1j}(1-\pi_{1j})) \\
&+ m_{21j} \ln(\rho_j(1-\pi_{1j}) + \pi_{1j}) + m_{22j} \ln(\rho_j \theta (1-\pi_{1j}) + \pi_{1j}) + m_{2+j} \ln(\pi_{1j}) \\
&- m_{+2j} \ln(\pi_{1j}(1-\theta) + \theta)^2.
\end{aligned}
$$

To take first-order derivatives:

$$
\begin{aligned}
\frac{\partial l_{j|\theta}}{\partial \pi_{1j}} =& \frac{n_{01j}}{\pi_{1j} - 1} + \frac{n_{02j}}{(\pi_{1j} - 1)\ (\pi_{1j} + \theta - \pi_{1j}\,\theta)} + \frac{n_{11j}}{\pi_{1j}} + \frac{n_{12j}\,\theta}{\pi_{1j}\ (\pi_{1j} + \theta - \pi_{1j}\,\theta)} \\
&- \frac{m_{01j}\ (2\,\pi_{1j} + \rho_j - 2\,\pi_{1j}\,\rho_j - 2)}{(\pi_{1j} - 1)\ (\pi_{1j}\,\rho_j - \pi_{1j} + 1)} + \frac{m_{02j}\ (2\,\theta + \pi_{1j}\,\rho_j - 2\,\pi_{1j}\,\theta - \rho_j\,\theta + \pi_{1j}\,\rho_j\,\theta)}{(\pi_{1j} - 1)\ (\theta + \pi_{1j}\,\rho_j - \pi_{1j}\,\theta)\ (\pi_{1j} + \theta - \pi_{1j}\,\theta)} \\
&+ \frac{m_{11j}\ (2\,\pi_{1j} - 1)}{\pi_{1j}\ (\pi_{1j} - 1)} + \frac{m_{12j}\ (\pi_{1j} - \theta + \pi_{1j}\,\theta)}{\pi_{1j}\ (\pi_{1j} - 1)\ (\pi_{1j} + \theta - \pi_{1j}\,\theta)} \\
&+ \frac{m_{21j}\ (2\,\pi_{1j} + \rho_j - 2\,\pi_{1j}\,\rho_j)}{\pi_{1j}\ (\pi_{1j} + \rho_j - \pi_{1j}\,\rho_j)} + \frac{m_{22j}\,\theta\ (2\,\pi_{1j} - \pi_{1j}\,\rho_j + \rho_j\,\theta - \pi_{1j}\,\rho_j\,\theta)}{\pi_{1j}\ (\pi_{1j} + \theta - \pi_{1j}\,\theta)\ (\pi_{1j} + \rho_j\,\theta - \pi_{1j}\,\rho_j\,\theta)} \\
\frac{\partial l_{j|\theta}}{\partial \rho_j} =& \frac{\pi_{1j}\,m_{01j}}{\pi_{1j}\,\rho_j - \pi_{1j} + 1} + \frac{\pi_{1j}\,m_{02j}}{\theta + \pi_{1j}\,\rho_j - \pi_{1j}\,\theta} + \frac{m_{11j}}{\rho_j - 1} + \frac{m_{12j}}{\rho_j - 1} \\
&- \frac{m_{21j}\ (\pi_{1j} - 1)}{\pi_{1j} + \rho_j - \pi_{1j}\,\rho_j} - \frac{m_{22j}\,\theta\ (\pi_{1j} - 1)}{\pi_{1j} + \rho_j\,\theta - \pi_{1j}\,\rho_j\,\theta} \\
\frac{\partial l_{j|\theta}}{\partial \theta} =& \frac{\pi_{1j}\,n_{02j}}{\theta\ (\pi_{1j} + \theta - \pi_{1j}\,\theta)} + \frac{n_{12j}\ (\pi_{1j} - 1)}{\pi_{1j} + \theta - \pi_{1j}\,\theta} \\
&+ \frac{\pi_{1j}\,m_{02j}\ (2\,\theta + \pi_{1j}\,\rho_j - 2\,\pi_{1j}\,\theta - \rho_j\,\theta + \pi_{1j}\,\rho_j\,\theta)}{\theta\ (\theta + \pi_{1j}\,\rho_j - \pi_{1j}\,\theta)\ (\pi_{1j} + \theta - \pi_{1j}\,\theta)} \\
&+ \frac{m_{12j}\ (\pi_{1j} - \theta + \pi_{1j}\,\theta)}{\theta\ (\pi_{1j} + \theta - \pi_{1j}\,\theta)} + \frac{m_{22j}\ (\pi_{1j} - 1)\ (2\,\pi_{1j} - \pi_{1j}\,\rho_j + \rho_j\,\theta - \pi_{1j}\,\rho_j\,\theta)}{(\pi_{1j} + \theta - \pi_{1j}\,\theta)\ (\pi_{1j} + \rho_j\,\theta - \pi_{1j}\,\rho_j\,\theta)}
\end{aligned}
$$

To take expectations of the negative second-order derivatives:

$$
\begin{aligned}
I_{11}^{(j)} =& E\left[-\frac{\partial^2 l_{j|\theta}}{\partial\theta^2}\right] = \frac{\pi_{1j}\, n_{0+j}\, (\pi_{1j}-1)^2}{(\pi_{1j}+\theta-\pi_{1j}\,\theta)^3} - \frac{\pi_{1j}\, n_{0+j}\ (\pi_{1j}-1)}{\theta\,(\pi_{1j}+\theta-\pi_{1j}\,\theta)^2} - \frac{\pi_{1j}\, n_{1+j}\, (\pi_{1j}-1)^2}{(\pi_{1j}+\theta-\pi_{1j}\,\theta)^3} \\
&+ \frac{\pi_{1j}\, m_{0+j}\, (\pi_{1j}-1)^2\ (2\,\theta+\pi_{1j}\,\rho_j-2\,\pi_{1j}\,\theta-\rho_j\,\theta+\pi_{1j}\,\rho_j\,\theta)}{(\pi_{1j}+\theta-\pi_{1j}\,\theta)^4} \\
&+ \frac{\pi_{1j}\, m_{0+j}\, (\pi_{1j}-1)^2\ (\rho_j-2)}{(\pi_{1j}+\theta-\pi_{1j}\,\theta)^3} \\
&- \frac{\pi_{1j}\, m_{0+j}\ (\pi_{1j}-1)\ (2\,\theta+\pi_{1j}\,\rho_j-2\,\pi_{1j}\,\theta-\rho_j\,\theta+\pi_{1j}\,\rho_j\,\theta)}{\theta\,(\pi_{1j}+\theta-\pi_{1j}\,\theta)^3} \\
&+ \frac{\pi_{1j}\, m_{0+j}\, (\pi_{1j}-1)^2\ (2\,\theta+\pi_{1j}\,\rho_j-2\,\pi_{1j}\,\theta-\rho_j\,\theta+\pi_{1j}\,\rho_j\,\theta)}{(\theta+\pi_{1j}\,\rho_j-\pi_{1j}\,\theta)\ (\pi_{1j}+\theta-\pi_{1j}\,\theta)^3} \\
&- \frac{2\,\pi_{1j}\, m_{1+j}\, (\pi_{1j}-1)^2\ (\rho_j-1)}{(\pi_{1j}+\theta-\pi_{1j}\,\theta)^3} - \frac{2\,\pi_{1j}\, m_{1+j}\, (\pi_{1j}-1)^2\ (\rho_j-1)\ (\pi_{1j}-\theta+\pi_{1j}\,\theta)}{(\pi_{1j}+\theta-\pi_{1j}\,\theta)^4} \\
&+ \frac{2\,\pi_{1j}\, m_{1+j}\ (\pi_{1j}-1)\ (\rho_j-1)\ (\pi_{1j}-\theta+\pi_{1j}\,\theta)}{\theta\,(\pi_{1j}+\theta-\pi_{1j}\,\theta)^3} \\
&- \frac{\pi_{1j}\, m_{2+j}\, (\pi_{1j}-1)^2\ (2\,\pi_{1j}-\pi_{1j}\,\rho_j+\rho_j\,\theta-\pi_{1j}\,\rho_j\,\theta)}{(\pi_{1j}+\theta-\pi_{1j}\,\theta)^4} + \frac{\pi_{1j}\, m_{2+j}\,\rho_j\, (\pi_{1j}-1)^2}{(\pi_{1j}+\theta-\pi_{1j}\,\theta)^3} \\
&- \frac{\pi_{1j}\, m_{2+j}\,\rho_j\, (\pi_{1j}-1)^2\ (2\,\pi_{1j}-\pi_{1j}\,\rho_j+\rho_j\,\theta-\pi_{1j}\,\rho_j\,\theta)}{(\pi_{1j}+\theta-\pi_{1j}\,\theta)^3\ (\pi_{1j}+\rho_j\,\theta-\pi_{1j}\,\rho_j\,\theta)} \\
I_{12}^{(j)} =& I_{21}^{(j)} = E\left[-\frac{\partial^2 l_{j|\theta}}{\partial\pi_{1j}\partial\theta}\right] = \frac{n_{0+j}\,\theta\ (\pi_{1j}-1)}{(\pi_{1j}+\theta-\pi_{1j}\,\theta)^3} - \frac{n_{1+j}}{(\pi_{1j}+\theta-\pi_{1j}\,\theta)^2} - \frac{n_{1+j}\,\theta\ (\pi_{1j}-1)}{(\pi_{1j}+\theta-\pi_{1j}\,\theta)^3} \\
&+ \frac{m_{0+j}\,\theta\ (\pi_{1j}-1)\ (\rho_j-2)}{(\pi_{1j}+\theta-\pi_{1j}\,\theta)^3} + \frac{m_{0+j}\,\theta\ (\pi_{1j}-1)\ (2\,\theta+\pi_{1j}\,\rho_j-2\,\pi_{1j}\,\theta-\rho_j\,\theta+\pi_{1j}\,\rho_j\,\theta)}{(\theta+\pi_{1j}\,\rho_j-\pi_{1j}\,\theta)\ (\pi_{1j}+\theta-\pi_{1j}\,\theta)^3} \\
&+ \frac{m_{0+j}\,\theta\ (\pi_{1j}-1)\ (2\,\theta+\pi_{1j}\,\rho_j-2\,\pi_{1j}\,\theta-\rho_j\,\theta+\pi_{1j}\,\rho_j\,\theta)}{(\pi_{1j}+\theta-\pi_{1j}\,\theta)^4} \\
&- \frac{2\, m_{1+j}\,\theta\ (\pi_{1j}-1)\ (\rho_j-1)}{(\pi_{1j}+\theta-\pi_{1j}\,\theta)^3} - \frac{2\, m_{1+j}\,\theta\ (\pi_{1j}-1)\ (\rho_j-1)\ (\pi_{1j}-\theta+\pi_{1j}\,\theta)}{(\pi_{1j}+\theta-\pi_{1j}\,\theta)^4} \\
&- \frac{m_{2+j}\ (2\,\pi_{1j}-\pi_{1j}\,\rho_j+\rho_j\,\theta-\pi_{1j}\,\rho_j\,\theta)}{(\pi_{1j}+\theta-\pi_{1j}\,\theta)^3} \\
&- \frac{m_{2+j}\,\theta\ (\pi_{1j}-1)\ (2\,\pi_{1j}-\pi_{1j}\,\rho_j+\rho_j\,\theta-\pi_{1j}\,\rho_j\,\theta)}{(\pi_{1j}+\theta-\pi_{1j}\,\theta)^4} \\
&+ \frac{m_{2+j}\,\rho_j\,\theta\ (\pi_{1j}-1)}{(\pi_{1j}+\theta-\pi_{1j}\,\theta)^3} - \frac{m_{2+j}\,\rho_j\,\theta\ (\pi_{1j}-1)\ (2\,\pi_{1j}-\pi_{1j}\,\rho_j+\rho_j\,\theta-\pi_{1j}\,\rho_j\,\theta)}{(\pi_{1j}+\theta-\pi_{1j}\,\theta)^3\ (\pi_{1j}+\rho_j\,\theta-\pi_{1j}\,\rho_j\,\theta)} \\
I_{13}^{(j)} =& I_{31}^{(j)} = E\left[-\frac{\partial^2 l_{j|\theta}}{\partial\theta\partial\rho_j}\right] = \frac{\pi_{1j}\, m_{0+j}\,\theta\,(\pi_{1j}-1)^2}{(\theta+\pi_{1j}\,\rho_j-\pi_{1j}\,\theta)\ (\pi_{1j}+\theta-\pi_{1j}\,\theta)^2} + \frac{\pi_{1j}\, m_{2+j}\ (\pi_{1j}-1)}{(\pi_{1j}+\theta-\pi_{1j}\,\theta)^2} \\
&+ \frac{\pi_{1j}\, m_{2+j}\,\rho_j\,\theta\,(\pi_{1j}-1)^2}{(\pi_{1j}+\theta-\pi_{1j}\,\theta)^2\ (\pi_{1j}+\rho_j\,\theta-\pi_{1j}\,\rho_j\,\theta)}
\end{aligned}
$$

$$
\begin{aligned}
I_{22}^{(j)} =& E\left[-\frac{\partial^2 l_{j|\theta}}{\partial \pi_{1j}^2}\right] = \frac{n_{0+j}\,\theta\,\left(\pi_{1j}-1\right)}{\left(\pi_{1j}+\theta-\pi_{1j}\,\theta\right)^3} - \frac{n_{1+j}}{\left(\pi_{1j}+\theta-\pi_{1j}\,\theta\right)^2} - \frac{n_{1+j}\,\theta\,\left(\pi_{1j}-1\right)}{\left(\pi_{1j}+\theta-\pi_{1j}\,\theta\right)^3} \\
&+ \frac{m_{0+j}\,\theta\,\left(\pi_{1j}-1\right)\,\left(2\,\theta+\pi_{1j}\,\rho_j-2\,\pi_{1j}\,\theta-\rho_j\,\theta+\pi_{1j}\,\rho_j\,\theta\right)}{\left(\pi_{1j}+\theta-\pi_{1j}\,\theta\right)^4} + \frac{m_{0+j}\,\theta\,\left(\pi_{1j}-1\right)\,\left(\rho_j-2\right)}{\left(\pi_{1j}+\theta-\pi_{1j}\,\theta\right)^3} \\
&+ \frac{m_{0+j}\,\theta\,\left(\pi_{1j}-1\right)\,\left(2\,\theta+\pi_{1j}\,\rho_j-2\,\pi_{1j}\,\theta-\rho_j\,\theta+\pi_{1j}\,\rho_j\,\theta\right)}{\left(\theta+\pi_{1j}\,\rho_j-\pi_{1j}\,\theta\right)\,\left(\pi_{1j}+\theta-\pi_{1j}\,\theta\right)^3} \\
&- \frac{2\,m_{1+j}\,\theta\,\left(\pi_{1j}-1\right)\,\left(\rho_j-1\right)}{\left(\pi_{1j}+\theta-\pi_{1j}\,\theta\right)^3} - \frac{2\,m_{1+j}\,\theta\,\left(\pi_{1j}-1\right)\,\left(\rho_j-1\right)\,\left(\pi_{1j}-\theta+\pi_{1j}\,\theta\right)}{\left(\pi_{1j}+\theta-\pi_{1j}\,\theta\right)^4} \\
&- \frac{m_{2+j}\,\left(2\,\pi_{1j}-\pi_{1j}\,\rho_j+\rho_j\,\theta-\pi_{1j}\,\rho_j\,\theta\right)}{\left(\pi_{1j}+\theta-\pi_{1j}\,\theta\right)^3} \\
&- \frac{m_{2+j}\,\theta\,\left(\pi_{1j}-1\right)\,\left(2\,\pi_{1j}-\pi_{1j}\,\rho_j+\rho_j\,\theta-\pi_{1j}\,\rho_j\,\theta\right)}{\left(\pi_{1j}+\theta-\pi_{1j}\,\theta\right)^4} \\
&+ \frac{m_{2+j}\,\rho_j\,\theta\,\left(\pi_{1j}-1\right)}{\left(\pi_{1j}+\theta-\pi_{1j}\,\theta\right)^3} - \frac{m_{2+j}\,\rho_j\,\theta\,\left(\pi_{1j}-1\right)\,\left(2\,\pi_{1j}-\pi_{1j}\,\rho_j+\rho_j\,\theta-\pi_{1j}\,\rho_j\,\theta\right)}{\left(\pi_{1j}+\theta-\pi_{1j}\,\theta\right)^3\,\left(\pi_{1j}+\rho_j\,\theta-\pi_{1j}\,\rho_j\,\theta\right)} \\
I_{23}^{(j)} =& I_{32}^{(j)} = E\left[-\frac{\partial^2 l_{j|\theta}}{\partial \pi_{1j}\partial \rho_j}\right] = m_{0+j}\,\left(2\,\pi_{1j}-1\right) + \frac{\pi_{1j}\,m_{0+j}\,\left(2\,\pi_{1j}+\rho_j-2\,\pi_{1j}\,\rho_j-2\right)}{\pi_{1j}\,\rho_j-\pi_{1j}+1} \\
&+ \frac{m_{0+j}\,\theta\,\left(\pi_{1j}-\theta+\pi_{1j}\,\theta\right)}{\left(\pi_{1j}+\theta-\pi_{1j}\,\theta\right)^3} - \frac{\pi_{1j}\,m_{0+j}\,\theta\,\left(2\,\theta+\pi_{1j}\,\rho_j-2\,\pi_{1j}\,\theta-\rho_j\,\theta+\pi_{1j}\,\rho_j\,\theta\right)}{\left(\theta+\pi_{1j}\,\rho_j-\pi_{1j}\,\theta\right)\,\left(\pi_{1j}+\theta-\pi_{1j}\,\theta\right)^3} \\
&+ m_{2+j}\,\left(2\,\pi_{1j}-1\right) - \frac{m_{2+j}\,\left(\pi_{1j}-1\right)\,\left(2\,\pi_{1j}+\rho_j-2\,\pi_{1j}\,\rho_j\right)}{\pi_{1j}+\rho_j-\pi_{1j}\,\rho_j} + \frac{m_{2+j}\,\theta\,\left(\pi_{1j}-\theta+\pi_{1j}\,\theta\right)}{\left(\pi_{1j}+\theta-\pi_{1j}\,\theta\right)^3} \\
&- \frac{m_{2+j}\,\theta^2\,\left(\pi_{1j}-1\right)\,\left(2\,\pi_{1j}-\pi_{1j}\,\rho_j+\rho_j\,\theta-\pi_{1j}\,\rho_j\,\theta\right)}{\left(\pi_{1j}+\theta-\pi_{1j}\,\theta\right)^3\,\left(\pi_{1j}+\rho_j\,\theta-\pi_{1j}\,\rho_j\,\theta\right)} \\
I_{33}^{(j)} =& E\left[-\frac{\partial^2 l_{j|\theta}}{\partial \rho_j^2}\right] = -\frac{{\pi_{1j}}^2\,m_{0+j}\,\left(\pi_{1j}-1\right)}{\pi_{1j}\,\rho_j-\pi_{1j}+1} - \frac{{\pi_{1j}}^2\,m_{0+j}\,\theta\,\left(\pi_{1j}-1\right)}{\left(\theta+\pi_{1j}\,\rho_j-\pi_{1j}\,\theta\right)\,\left(\pi_{1j}+\theta-\pi_{1j}\,\theta\right)^2} \\
&+ \frac{2\,\pi_{1j}\,m_{1+j}\,\left(\pi_{1j}-1\right)}{\rho_j-1} + \frac{2\,\pi_{1j}\,m_{1+j}\,\theta\,\left(\pi_{1j}-1\right)}{\left(\rho_j-1\right)\,\left(\pi_{1j}+\theta-\pi_{1j}\,\theta\right)^2} \\
&+ \frac{\pi_{1j}\,m_{2+j}\,\left(\pi_{1j}-1\right)^2}{\pi_{1j}+\rho_j-\pi_{1j}\,\rho_j} + \frac{\pi_{1j}\,m_{2+j}\,\theta^2\,\left(\pi_{1j}-1\right)^2}{\left(\pi_{1j}+\theta-\pi_{1j}\,\theta\right)^2\,\left(\pi_{1j}+\rho_j\,\theta-\pi_{1j}\,\rho_j\,\theta\right)}
\end{aligned}
$$

Test Statistics Derivation

Since

$$\boldsymbol{I}_j^{-1} = \frac{1}{D_j}\begin{pmatrix} I_{23}^{(j)}I_{23}^{(j)} - I_{22}^{(j)}I_{33}^{(j)} & I_{12}^{(j)}I_{33}^{(j)} - I_{13}^{(j)}I_{23}^{(j)} & I_{13}^{(j)}I_{22}^{(j)} - I_{12}^{(j)}I_{23}^{(j)} \\ I_{12}^{(j)}I_{33}^{(j)} - I_{13}^{(j)}I_{23}^{(j)} & I_{13}^{(j)}I_{13}^{(j)} - I_{11}^{(j)}I_{33}^{(j)} & I_{11}^{(j)}I_{23}^{(j)} - I_{12}^{(j)}I_{13}^{(j)} \\ I_{13}^{(j)}I_{22}^{(j)} - I_{12}^{(j)}I_{23}^{(j)} & I_{11}^{(j)}I_{23}^{(j)} - I_{12}^{(j)}I_{13}^{(j)} & I_{12}^{(j)}I_{12}^{(j)} - I_{11}^{(j)}I_{22}^{(j)} \end{pmatrix},$$

where $D_j = I_{33}^{(j)}(I_{12}^{(j)})^2 - 2I_{12}^{(j)}I_{13}^{(j)}I_{23}^{(j)} + I_{22}^{(j)}(I_{13}^{(j)})^2 + I_{11}^{(j)}(I_{23}^{(j)})^2 - I_{11}^{(j)}I_{22}^{(j)}I_{33}^{(j)}$, we then have:

$$T_{SC} = \sum_{j=1}^{J}\boldsymbol{U}_j\boldsymbol{I}_j^{-1}(\tilde{\boldsymbol{\alpha}})\boldsymbol{U}_j^T = \sum_{j=1}^{J}(\frac{\partial l_{j|\theta}}{\partial\theta})^2\frac{1}{D_j}(I_{23}^{(j)}I_{23}^{(j)} - I_{22}^{(j)}I_{33}^{(j)})^2|_{(\theta=\tilde{\theta},\pi_{1j}=\tilde{\pi}_{1j},\rho_j=\tilde{\rho}_j)}.$$

Wald Test

Information Matrix Derivation

Consider the log likelihood function within a stratum under no constraint, we have:

$$\begin{aligned} l_j(\pi_{1j},\pi_{2j},\rho_j) = \sum_{i=1}^{2}[&n_{0ij}\log(1-\pi_{ij})) + n_{1ij}\log\pi_{ij} + m_{0ij}\log((1-\pi_{ij})(\rho\pi_{ij}-\pi_{ij}+1)) \\ &+ m_{1ij}\log(2\pi_{ij}(1-\pi_{ij})(1-\rho_j)) + m_{2ij}\log(\pi_{ij}(\rho_j+\pi_{ij}-\rho_j\pi_{ij}))]. \end{aligned}$$

To take first-order derivatives:

$$\begin{aligned} \frac{\partial l_j}{\partial\pi_{1j}} =& \frac{n_{01j}}{\pi_{1j}-1} + \frac{n_{11j}}{\pi_{1j}} \\ &- \frac{m_{01j}\ (2\,\pi_{1j}+\rho_j-2\,\pi_{1j}\,\rho_j-2)}{(\pi_{1j}-1)\ (\pi_{1j}\,\rho_j-\pi_{1j}+1)} + \frac{m_{11j}\ (2\,\pi_{1j}-1)}{\pi_{1j}\ (\pi_{1j}-1)} + \frac{m_{21j}\ (2\,\pi_{1j}+\rho_j-2\,\pi_{1j}\,\rho_j)}{\pi_{1j}\ (\pi_{1j}+\rho_j-\pi_{1j}\,\rho_j)} \\ \frac{\partial l_j}{\partial\pi_{2j}} =& \frac{n_{02j}}{\pi_{2j}-1} + \frac{n_{12j}}{\pi_{2j}} \\ &- \frac{m_{02j}\ (2\,\pi_{2j}+\rho_j-2\,\pi_{2j}\,\rho_j-2)}{(\pi_{2j}-1)\ (\pi_{2j}\,\rho_j-\pi_{2j}+1)} + \frac{m_{12j}\ (2\,\pi_{2j}-1)}{\pi_{2j}\ (\pi_{2j}-1)} + \frac{m_{22j}\ (2\,\pi_{2j}+\rho_j-2\,\pi_{2j}\,\rho_j)}{\pi_{2j}\ (\pi_{2j}+\rho_j-\pi_{2j}\,\rho_j)} \\ \frac{\partial l_j}{\partial\rho_j} =& \frac{\pi_{1j}\,m_{01j}}{\pi_{1j}\,\rho_j-\pi_{1j}+1} + \frac{\pi_{2j}\,m_{02j}}{\pi_{2j}\,\rho_j-\pi_{2j}+1} + \frac{m_{11j}}{\rho_j-1} + \frac{m_{12j}}{\rho_j-1} \\ &- \frac{\pi_{1j}\,m_{21j}\ (\pi_{1j}-1)}{{\pi_{1j}}^2-\pi_{1j}\,\rho_j\ (\pi_{1j}-1)} - \frac{\pi_{2j}\,m_{22j}\ (\pi_{2j}-1)}{{\pi_{2j}}^2-\pi_{2j}\,\rho_j\ (\pi_{2j}-1)} \end{aligned}$$

To take expectations of the negative second-order derivatives:

$$
\begin{aligned}
I_{11}^{(j)} =& E\left[-\frac{\partial^2 l_j}{\partial \pi_{1j}^2}\right] = -\frac{n_{0+j}}{\pi_{1j}-1} + \frac{n_{1+j}}{\pi_{1j}} \\
&+ 2\,m_{0+j}\,\left(\rho_j-1\right) + \frac{m_{0+j}\,\left(2\,\pi_{1j}+\rho_j-2\,\pi_{1j}\,\rho_j-2\right)}{\pi_{1j}-1} \\
&+ \frac{m_{0+j}\,\left(\rho_j-1\right)\,\left(2\,\pi_{1j}+\rho_j-2\,\pi_{1j}\,\rho_j-2\right)}{\pi_{1j}\,\rho_j-\pi_{1j}+1} \\
&- 4\,m_{1+j}\,\left(\rho_j-1\right) + \frac{2\,m_{1+j}\,\left(2\,\pi_{1j}-1\right)\,\left(\rho_j-1\right)}{\pi_{1j}} + \frac{2\,m_{1+j}\,\left(2\,\pi_{1j}-1\right)\,\left(\rho_j-1\right)}{\pi_{1j}-1} \\
&+ 2\,m_{2+j}\,\left(\rho_j-1\right) + \frac{m_{2+j}\,\left(2\,\pi_{1j}+\rho_j-2\,\pi_{1j}\,\rho_j\right)}{\pi_{1j}} \\
&- \frac{m_{2+j}\,\left(\rho_j-1\right)\,\left(2\,\pi_{1j}+\rho_j-2\,\pi_{1j}\,\rho_j\right)}{\pi_{1j}+\rho_j-\pi_{1j}\,\rho_j} \\
I_{12}^{(j)} &= I_{21}^{(j)} = E\left[-\frac{\partial^2 l_j}{\partial \pi_{1j}\partial \pi_{2j}}\right] = 0 \\
I_{13}^{(j)} =& I_{31}^{(j)} = E\left[-\frac{\partial^2 l_j}{\partial \pi_{1j}\partial \rho_j}\right] = m_{0+j}\,\left(2\,\pi_{1j}-1\right) + \frac{\pi_{1j}\,m_{0+j}\,\left(2\,\pi_{1j}+\rho_j-2\,\pi_{1j}\,\rho_j-2\right)}{\pi_{1j}\,\rho_j-\pi_{1j}+1} \\
&+ m_{2+j}\,\left(2\,\pi_{1j}-1\right) - \frac{m_{2+j}\,\left(\pi_{1j}-1\right)\,\left(2\,\pi_{1j}+\rho_j-2\,\pi_{1j}\,\rho_j\right)}{\pi_{1j}+\rho_j-\pi_{1j}\,\rho_j}
\end{aligned}
$$

$$
\begin{aligned}
I_{22}^{(j)} =& E\left[-\frac{\partial^2 l_j}{\partial \pi_{2j}^2}\right] = -\frac{n_{0+j}}{\pi_{2j}-1} + \frac{n_{1+j}}{\pi_{2j}} \\
&+ 2\,m_{0+j}\,\left(\rho_j-1\right) + \frac{m_{0+j}\,\left(2\,\pi_{2j}+\rho_j-2\,\pi_{2j}\,\rho_j-2\right)}{\pi_{2j}-1} \\
&+ \frac{m_{0+j}\,\left(\rho_j-1\right)\,\left(2\,\pi_{2j}+\rho_j-2\,\pi_{2j}\,\rho_j-2\right)}{\pi_{2j}\,\rho_j-\pi_{2j}+1} \\
&- 4\,m_{1+j}\,\left(\rho_j-1\right) + \frac{2\,m_{1+j}\,\left(2\,\pi_{2j}-1\right)\,\left(\rho_j-1\right)}{\pi_{2j}} + \frac{2\,m_{1+j}\,\left(2\,\pi_{2j}-1\right)\,\left(\rho_j-1\right)}{\pi_{2j}-1} \\
&+ 2\,m_{2+j}\,\left(\rho_j-1\right) + \frac{m_{2+j}\,\left(2\,\pi_{2j}+\rho_j-2\,\pi_{2j}\,\rho_j\right)}{\pi_{2j}} \\
&- \frac{m_{2+j}\,\left(\rho_j-1\right)\,\left(2\,\pi_{2j}+\rho_j-2\,\pi_{2j}\,\rho_j\right)}{\pi_{2j}+\rho_j-\pi_{2j}\,\rho_j} \\
I_{23}^{(j)} =& I_{32}^{(j)} = E\left[-\frac{\partial^2 l_j}{\partial \pi_{2j}\partial \rho_j}\right] = m_{0+j}\,\left(2\,\pi_{2j}-1\right) + \frac{\pi_{2j}\,m_{0+j}\,\left(2\,\pi_{2j}+\rho_j-2\,\pi_{2j}\,\rho_j-2\right)}{\pi_{2j}\,\rho_j-\pi_{2j}+1} \\
&+ m_{2+j}\,\left(2\,\pi_{2j}-1\right) - \frac{m_{2+j}\,\left(\pi_{2j}-1\right)\,\left(2\,\pi_{2j}+\rho_j-2\,\pi_{2j}\,\rho_j\right)}{\pi_{2j}+\rho_j-\pi_{2j}\,\rho_j} \\
I_{33}^{(j)} =& E\left[-\frac{\partial^2 l_j}{\partial \rho_j^2}\right] = -\frac{{\pi_{1j}}^2\,m_{0+j}\,\left(\pi_{1j}-1\right)}{\pi_{1j}\,\rho_j-\pi_{1j}+1} - \frac{{\pi_{2j}}^2\,m_{0+j}\,\left(\pi_{2j}-1\right)}{\pi_{2j}\,\rho_j-\pi_{2j}+1} + \frac{2\,\pi_{1j}\,m_{1+j}\,\left(\pi_{1j}-1\right)}{\rho_j-1} \\
&+ \frac{2\,\pi_{2j}\,m_{1+j}\,\left(\pi_{2j}-1\right)}{\rho_j-1} + \frac{\pi_{1j}\,m_{2+j}\,{\left(\pi_{1j}-1\right)}^2}{\pi_{1j}+\rho_j-\pi_{1j}\,\rho_j} + \frac{\pi_{2j}\,m_{2+j}\,{\left(\pi_{2j}-1\right)}^2}{\pi_{2j}+\rho_j-\pi_{2j}\,\rho_j}
\end{aligned}
$$

Test Statistics Derivation

Consider

$$\boldsymbol{I}_{\boldsymbol{\beta}} \equiv \begin{pmatrix} \boldsymbol{I}_1 & \boldsymbol{0} & \cdots & \boldsymbol{0} \\ \boldsymbol{0} & \boldsymbol{I}_2 & \cdots & \boldsymbol{0} \\ \vdots & \vdots & \ddots & \vdots \\ \boldsymbol{0} & \boldsymbol{0} & \cdots & \boldsymbol{I}_J \end{pmatrix},$$

where

$$\boldsymbol{I}_j = \begin{pmatrix} I_{11}^{(j)} & I_{12}^{(j)} & I_{13}^{(j)} \\ I_{21}^{(j)} & I_{22}^{(j)} & I_{23}^{(j)} \\ I_{31}^{(j)} & I_{32}^{(j)} & I_{33}^{(j)} \end{pmatrix}.$$

Thus,

$$\begin{aligned} T_W &= (\boldsymbol{C}\hat{\boldsymbol{\delta}})^T Var^{-1}(\boldsymbol{C}\hat{\boldsymbol{\delta}})(\boldsymbol{C}\hat{\boldsymbol{\delta}}) \\ &= (\hat{\boldsymbol{\delta}}^T \boldsymbol{C}^T)(\boldsymbol{C}\Delta g \boldsymbol{I}_{\hat{\boldsymbol{\beta}}}{}^{-1} \Delta g^T \boldsymbol{C}^T)^{-1}(\boldsymbol{C}\hat{\boldsymbol{\delta}})|_{\hat{\boldsymbol{\beta}}=(\hat{\pi}_{11},\hat{\pi}_{21},\hat{\rho}_1,\ldots,\hat{\pi}_{1J},\hat{\pi}_{2J},\hat{\rho}_J)}, \end{aligned}$$
